# Novel mRNA-specific effects of ribosome drop-off on translation rate and polysome profile

**DOI:** 10.1371/journal.pcbi.1005555

**Published:** 2017-05-30

**Authors:** Pierre Bonnin, Norbert Kern, Neil T. Young, Ian Stansfield, M. Carmen Romano

**Affiliations:** 1 Institute for Complex Systems and Mathematical Biology, Physics Department, University of Aberdeen, Aberdeen, UK; 2 Institute of Medical Sciences, University of Aberdeen, Foresterhill, Aberdeen, UK; 3 Université Montpellier 2, Laboratoire Charles Coulomb UMR 5221, Montpellier, France; Rutgers University, UNITED STATES

## Abstract

The well established phenomenon of ribosome drop-off plays crucial roles in translational accuracy and nutrient starvation responses during protein translation. When cells are under stress conditions, such as amino acid starvation or aminoacyl-tRNA depletion due to a high level of recombinant protein expression, ribosome drop-off can substantially affect the efficiency of protein expression. Here we introduce a mathematical model that describes the effects of ribosome drop-off on the ribosome density along the mRNA and on the concomitant protein synthesis rate. Our results show that ribosome premature termination may lead to non-intuitive ribosome density profiles, such as a ribosome density which increases from the 5’ to the 3’ end. Importantly, the model predicts that the effects of ribosome drop-off on the translation rate are mRNA-specific, and we quantify their resilience to drop-off, showing that the mRNAs which present ribosome queues are much less affected by ribosome drop-off than those which do not. Moreover, among those mRNAs that do not present ribosome queues, resilience to drop-off correlates positively with the elongation rate, so that sequences using fast codons are expected to be less affected by ribosome drop-off. This result is consistent with a genome-wide analysis of *S. cerevisiae*, which reveals that under favourable growth conditions mRNAs coding for proteins involved in the translation machinery, known to be highly codon biased and using preferentially fast codons, are highly resilient to ribosome drop-off. Moreover, in physiological conditions, the translation rate of mRNAs coding for regulatory, stress-related proteins, is less resilient to ribosome drop-off. This model therefore allows analysis of variations in the translational efficiency of individual mRNAs by accounting for the full range of known ribosome behaviours, as well as explaining mRNA-specific variations in ribosome density emerging from ribosome profiling studies.

## Author summary

Ribosomes often drop off the messenger RNA (mRNA) molecules during the process of translation. This phenomenon occurs under normal conditions and it is especially aggravated if the cell is subject to stress. In this paper we show that protein production rates and polysome profiles along the mRNA can be strongly affected by ribosome drop-off. It can lead to a non-intuitive increasing ribosome density profile from the 5’ to 3’ end, as well as coexistence between low and high ribosomal density along the mRNA. Importantly, we show that the repercussions of ribosome drop-off are mRNA-dependent, with the specific codon configuration and composition determining their resilience to ribosome drop-off effects.

## Introduction

Translational control of gene expression is acknowledged to be a key regulatory control point governing the relationship between mRNA expression levels, and the levels of the encoded polypeptide [1–4]. The expression levels of a large number of genes are regulated at the translational level [5, 6]. Recent advances in measurement techniques, such as ribosome profiling by deep sequencing of ribosome-protected mRNA fragments [7–10], now permit a much more detailed study of translational dynamics under a series of different cellular conditions. That understanding has been considerably advanced by the development of a range of mathematical models of translation, which have been utilised alongside experiments for more systematic and formal hypothesis testing [11–17].

One important aspect which has been considered only in a few models [18–22] is the fact that ribosomes are subject to premature translational abandonment, so-called ribosome drop-off events. These can occur at non-stop codons anywhere in the open reading frame, and represent an energetically expensive form of translational error which results in the proteolytic degradation of the non-functional and partly made polypeptide. For this reason, evolutionary selection has presumably acted to minimise this form of translational error [23–25]. However, premature ribosome abandonment of translation can also be a targeted ‘emergency’ response triggered specifically by long pauses at any given codon. The existence of mechanisms to resolve such stalled complexes is necessary because cells devote a large percentage of their energy resources to produce ribosomes and must therefore prevent them from becoming locked in non-productive stalled queues [24]. Such pauses could be caused for example by depletion of aminoacyl-tRNA [20, 21], the incorporation of an amino acid from a non-cognate tRNA [26], the formation of a ‘non-stop’ translation complex or the presence of specific amino acid sequence motifs in the nascent polypeptide [24]. Rescuing ribosomes that have stalled in this way requires specific *trans*-acting factors, for example tmRNA and ArfA in bacteria, and Dom34 in eukaryotic systems [25, 27, 28].

Recently, Sin et al [29] have introduced a novel method to analyse ribosome profiling data, and they have shown that ribosome drop-off rates are statistically significant under physiological conditions, with an estimated rate of 10^−4^ events per codon in *E. coli*, in accordance with [30]. Using the average speed of a ribosome of roughly 10 codons/s [31] this corresponds to a rate of *γ* = 10^−3^ *s*^−1^ at which the ribosomes drop off the mRNA lattice prematurely. Importantly, Sin et al. have also shown that drop-off rates can substantially increase under stress conditions, e.g. under amino acid starvation.

Here we show that taking into account the effects of ribosome drop-off during translation is crucial for modelling and correctly interpreting ribosome profiling data and, consequently, the derived estimates of translation rates. We present a mathematical model that describes ribosome drop-off and allows the derivation of analytical results that provide insight into the effects of premature termination of translation. It also allows gene-wide simulations to be performed to extrapolate the theoretical results to realistic mRNA sequences. Previous mathematical models including premature termination of ribosomes have focussed on different aspects of translation, rather than on ribosome drop-off alone. For example, in [18] and [19] ribosome interactions along the mRNA have been neglected, and the models presented in [20, 21] are mainly computational and do not provide analytical results. The recent work presented in [22] uses a related approach to the one taken in this paper, but it considers both attachment and detachment of particles, and focusses on the proof of existence of a unique equilibrium in the model solution.

Our model is based on the Totally Asymmetric Simple Exclusion Process (TASEP), an extensively studied model for transport processes in non-equilibrium statistical physics [32–35]. This model, introduced initially to describe translation [36], predicts the current of particles along a one-dimensional lattice, where particles represent ribosomes, and the lattice corresponds to the mRNA strand. Particles bind to the first site of the lattice at rate *α*, hop stochastically from one site of the lattice to the next with rate *k*, provided that the next site is free, and leave the lattice from the last site at rate *β*. The lattice sites thus represent the codons of the mRNA, and the hopping rate *k* can be estimated based on the concentration of the corresponding tRNAs, *i.e.* the molecules that deliver the amino acids to the ribosome to assemble the polypeptide chain [37]. In this paper we incorporate ribosome drop-off into this model by allowing particles to leave the lattice at any codon at a certain rate *γ*, as in [38]. We then analyse the effects of this drop-off rate on the ribosome current and density profiles along the mRNA, and show how this affects the rate at which proteins are being produced. We show that ribosome profiles can be substantially modified due to the drop-off and that, under circumstances relevant in physiological and stress situations, non-intuitive density profiles may result in which the ribosome concentration increases along the segment. Analogously to the case of TASEP with Langmuir kinetics [38, 39], coexistence of low and high ribosome density domains within the same mRNA can be obtained, separated by a localised matching region known as a domain wall.

In principle, the rate at which a ribosome hops along the mRNA sequence is non-uniform, since each codon is translated at a rate proportional to the concentration of its cognate tRNA, and codons are therefore defined as fast- or slow-translated. Importantly, the translation rate is not only affected by the number of such slow codons, but also by the way in which they are arranged along the mRNA [17, 37]. In general terms, an mRNA which has a slow codon or a stretch of slow codons in the middle or towards the 3’ end, can give rise to a queue of ribosomes if the translation initiation rate is larger than the elongation rate associated to those slow codons. In contrast, if an mRNA does not have slow codons, or has slow codons at the 5’ end, it cannot sustain significant ribosomal queue formation, since any backlog of ribosomes will be limited to a small portion of the sequence. These two main types of mRNAs (able or unable to sustain ribosome queues upon an increase in initiation rate) exhibit different ribosome traffic dynamics, and this mRNA classification has been shown to significantly correlate with Gene Ontology (GO): mRNAs able to sustain ribosome queues for large initiation rates are overrepresented in the regulatory proteins GO class, such as transcription factors, whereas mRNAs that cannot give rise to ribosome queues predominantly code for ribosomal proteins or proteins involved in the translation machinery (see [17] for a more detailed discussion). Importantly, the model presented here predicts that the translation rates of mRNAs able to sustain ribosome queues for large values of the initiation rate are much less affected by ribosome drop-off than those mRNAs which cannot present ribosome queues.

The model also makes a second important prediction with physiological relevance, that for those mRNAs not sustaining a queue of ribosomes, their generally higher translation rates render them more resilient to ribosome drop-off. In fact, a *S. cerevisiae* genome-wide analysis of the effects of ribosome drop-off on translation rate under physiological conditions, together with a Gene Ontology analysis, reveals that highly codon biased, rapidly translated mRNAs coding predominantly for proteins involved in translation are highly resilient to drop-off, in contrast to mRNAs coding for regulatory proteins.

This paper is organised as follows: we introduce the models which our analysis is based on in ‘Materials and Methods’, and then we derive some mathematical results for the ribosome drop-off model in the first part of the ‘Results’ section. The last 5 subsections of the manuscript deal with the application of the theoretical results to more realistic biological situations, including a genome-wide analysis of the effects of ribosome drop-off in *S. cerevisiae*. We therefore point the reader interested in the biological implications of our analysis to first focus on the last 4 subsections from ‘Results’, as well as the ‘Discussion’ section.

## Materials and methods

We now introduce the models which our analysis is based on, and present analytical results for an idealised representation. This will provide guidance for our analysis of the ribosome drop-off model, as well as for the study of more realistic mRNA sequences in the ‘Results’ section.

### Stochastic model of ribosome drop-off

#### Review of TASEP

The original TASEP model [36] consists of a one-dimensional lattice of *N* sites spanning a length *L*. Particles hop stochastically along this lattice, from site to site. The particles have hard sphere properties, and therefore can neither occupy the same site, nor can they overtake each other. The dynamics are defined through the microscopic behaviour of particles, which can

1. enter at a rate *α*, if site 1 is unoccupied (left boundary),
2. hop from site *i* to site *i* + 1 at a rate *k*, if site *i* + 1 is empty (bulk),
3. exit the lattice at a rate *β* from site *N* (right boundary).

The language above, used commonly, is adapted to a transport problem in which a current of particles flows from the left (upstream) boundary to the right (downstream) boundary. In the context of translation, the lattice sites correspond to codons of the mRNA strand, along which ribosomes advance stochastically as they translate the sequence, from the left (5’) end to the right (3’) end. The microscopic rates correspond to initiation (*α*), elongation (*k*) and termination (*β*).

The state of the system is parameterised in terms of its occupation numbers *n*_*i*_(*t*) for each site *i* of the lattice, so that *n*_*i*_(*t*) is either 1 (occupied) or 0 (empty) at time *t*. We can then write master equations for the time-averaged occupation numbers *ρ*_*i*_ = 〈*n_i_*(*t*)〉_*t*_, which in the mean-field approximation (neglecting correlations between the sites) is given by

$$\frac{d\rho_{1}}{dt}=\alpha \left(1-\rho_{1}\right)-k\rho_{1}\left(1-\rho_{2}\right)$$ (1)

$$\frac{d\rho_{i}}{dt}=k\rho_{i-1}\left(1-\rho_{i}\right)-k\rho_{i}\left(1-\rho_{i+1}\right),\;\;\text{for}\;\;i=2,...,N-1$$ (2)

$$\frac{d\rho_{N}}{dt}=k\rho_{N-1}\left(1-\rho_{N}\right)-\beta \rho_{N}.$$ (3)

(Note that the Ribosome Flow Model (RFM) [40] analyses the solutions of the mean-field approximation of TASEP-like models of translation.) We shall assume a steady state for all *ρ*_*i*_. We obtain the current of particles at a given position (number of particles going from site *i* to site *i* + 1 per unit time) as

$$J=k\rho_{i}\left(1-\rho_{i+1}\right).$$ (4)

As the number of particles is conserved along the lattice, this current does not depend on the site index *i*. Notice that every time a particle (ribosome) advances by one site (codon), another element is added to the nascent chain of amino acids. Thus the steady-state current is proportional to the amino acid incorporation rate and, in particular, the current at the 3’ end (right hand side) is proportional to the number of fully formed proteins produced per unit time, i.e. to the translation rate.

Four different phases [42–44] can be distinguished, depending on the relative values of *α*, *β* and *k*, and characterised by the density of particles along the lattice:

- low density (LD): if *α* < *β* and *α* < *k*/2, the entry rate limits the dynamics. The density in the bulk is *ρ* = *α*/*k* and *J* = *α*(1 − *α*/*k*).
- high density (HD): if *β* < *α* and *β* < *k*/2, the exit rate is the limiting step of the process. The density in the bulk is given by *ρ* = 1 − *β*/*k* and *J* = *β*(1 − *β*/*k*).
- maximal current (MC): if *α*, *β* > *k*/2 the system is limited by the bulk hopping rate *k*, and it achieves the maximal possible current *J* = *k*/4 at a density *ρ* = 1/2.
- shock phase (SP): in the particular case *α* = *β* and both *α*, *β* < *k*/2, both low density and high density regions coexist within the lattice. They are then separated by a domain wall which is non-localised, as it can be shown to diffuse randomly throughout the entire lattice [45].

**Fig 1:**
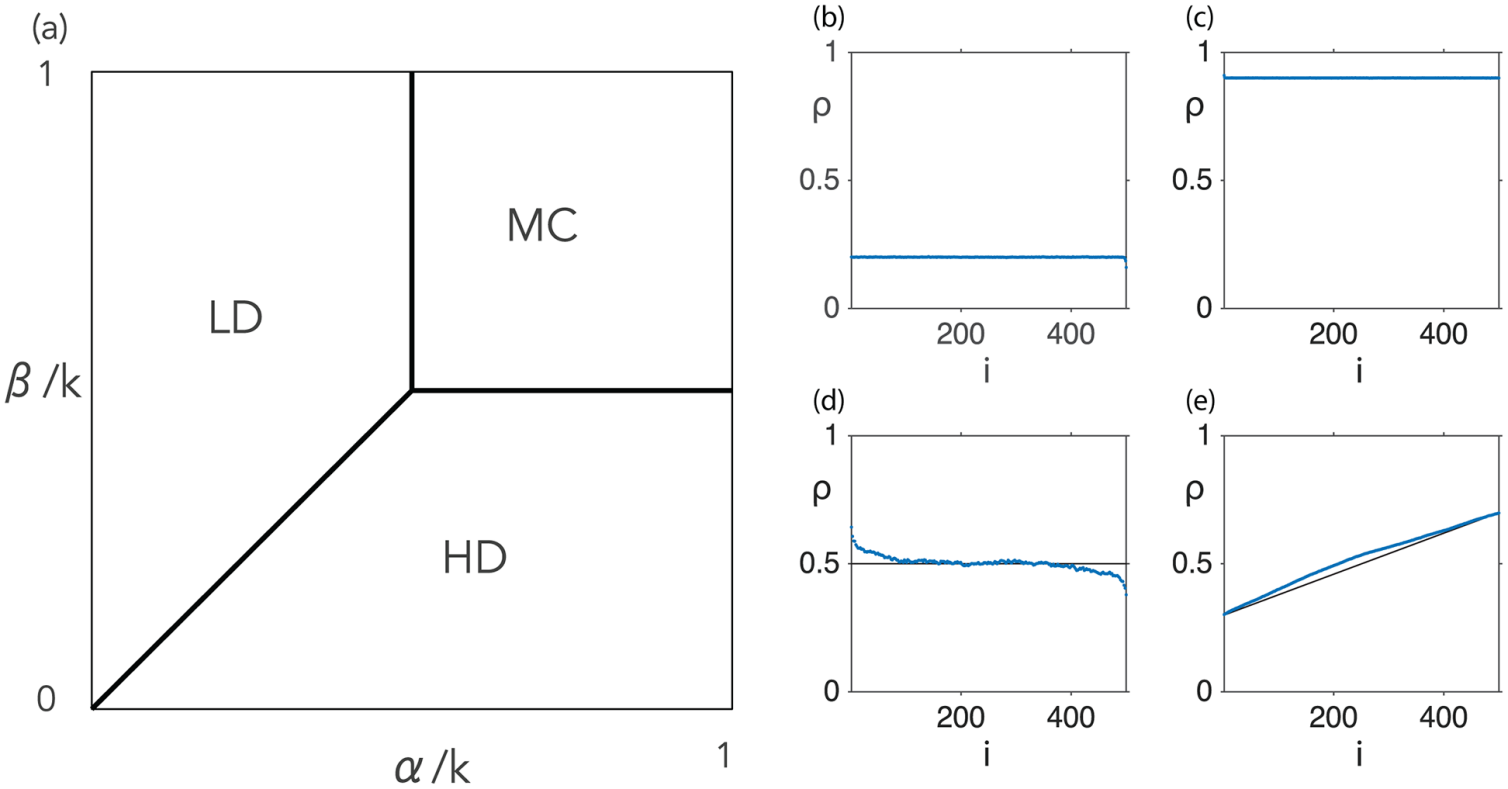
Main characteristics of the TASEP model. (a) Phase diagram. A low density (LD) phase, with density *ρ* < 1/2, arises when the current is limited by the input rate *α*. Symmetrically, a high density (HD) phase, with *ρ* > 1/2 is observed when the flow is governed by the output rate *β*. When neither is limiting, in the ‘maximal current’ phase (MC), the current is as large as the hopping process in the bulk permits (*J* = *k*/4 for *ρ* = 1/2). The line separating the LD and HD phases is where a shock phase (SP) is observed, where LD and HD zones coexist along the lattice. In this case the (few) sites corresponding to the interface between these these two zones is known as the ‘domain wall’. (b)-(e) Density profiles *ρ*_*i*_ of the original TASEP for a lattice of *N* = 500 sites and hopping rate *k* = 1. (b): LD regime: *α* = 0.2, *β* = 1; (c): HD regime: *α* = 1, *β* = 0.1; (d): MC regime: *α* = *β* = 0.5; (e): SP regime: *α* = *β* = 0.3. The solid black line represents the analytical solution from the mean-field approximation, and the blue points correspond to numerical simulations using the Gillespie algorithm [41].

All these phases are represented in Fig 1a, which constitutes the ‘phase diagram’ of this simplest version of the TASEP. These results can be most easily obtained from the mean-field approximation, but they are known to become exact in the limit of an infinitely long lattice [46].

The corresponding characteristic density profiles (time-averaged occupation numbers along the lattice) are illustrated in Fig 1b–1e. It is thus important to note that the densities given in (i)-(iv) above apply to the *bulk* of the lattice. Close to the boundaries deviations from these values arise, since the entry and exit rates imply constraints on the boundary sites which must be matched by the density profile. The deviation within these boundary layers can be estimated from the mean-field approach by considering current conservation along the lattice [46].

#### Incorporation of ribosome drop-off

**Fig 2:**
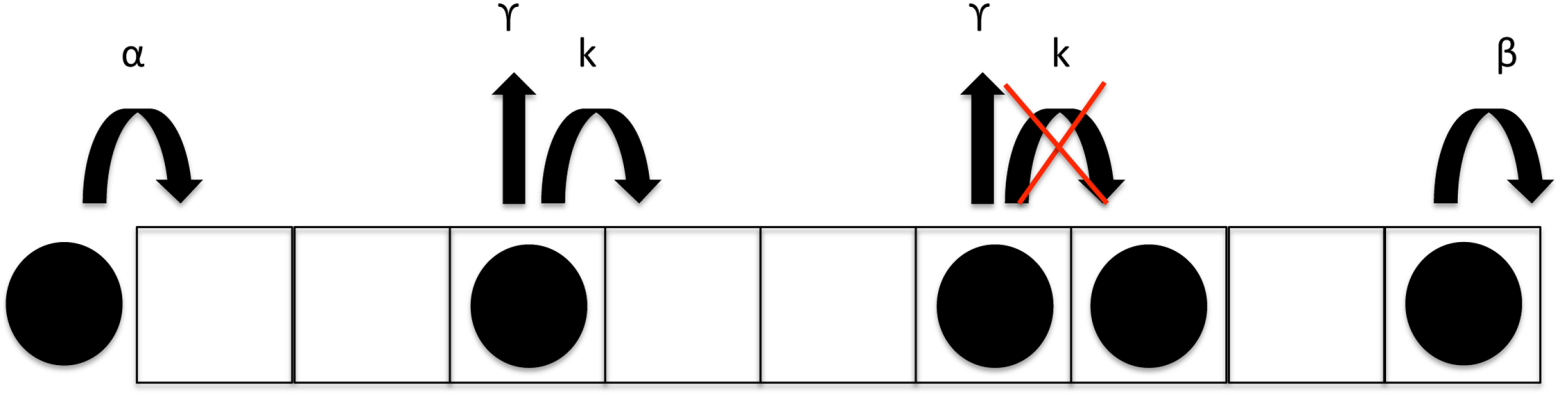
Illustration of the ribosome drop-off model. Ribosomes bind to the first codon of the mRNA with rate *α* and leave the lattice representing the mRNA strand at the stop codon, with rate *β*. Throughout the mRNA lattice they can either hop to the next codon, if it is free, with rate *k*, or drop off the lattice with rate *γ*.

As discussed in the introduction, we set out to study the effect of ribosome drop-off along the mRNA, and in particular we wish to predict the effects of ribosome drop-off on both ribosome density profiles and translation rate. We adopt the TASEP model and include an additional detachment rate *γ* at every site *i* of the lattice (see illustration in Fig 2). Hence, ribosomes can either hop to the next codon at rate *k*, given the corresponding site is free, or drop off the lattice at rate *γ*. We shall show that adding this reaction rate can have a significant impact on the translation rate. Before addressing the question of realistic sequences, we first establish insight into how the drop-off rate influences the traffic dynamics within the model outlined above. We derive analytical expressions for the density profiles in the next section, which we then compare to stochastic simulations of the process, performed using the Gillespie algorithm [41].

This first version of the model, based on a uniform drop-off rate, is directly related to the TASEP model with Langmuir kinetics (TASEP-LK), which considers a TASEP of particles which can both bind to and unbind from the lattice at any site [38, 39]. In that case a Langmuir-like equilibrium is established with the environment, and the implications on the current are well understood. However, the discussion in [38, 39] is based on the case where binding dominates with respect to unbinding, a case of interest in the context of transport by molecular motors but not representative of the biology of translation, where only drop-off of particles occurs. Although a mapping between the two cases can be exploited based on mathematical symmetries, it will be important in the following to establish direct intuition for the drop-off problem.

We therefore pinpoint the effects of ribosome drop-off on translation dynamics, which are not obvious in [38, 39]. As a consequence, the analytical expressions derived in our paper are different from the ones in [38, 39], and provide an interpretation for the effects of ribosome drop-off on translation. We therefore adapt our vocabulary to the problem of translation and analyse in detail how different types of mRNAs are affected by ribosome drop-off.

As to the phases, we shall continue to refer to them as LD, HD and MC, even in the presence of drop-off. We do so despite the fact that their characteristics are impacted by drop-off: indeed, since the particle current is no longer conserved along the segment, there is no reason for the density to be constant along the lattice, even within the bulk. In particular, the MC phase no longer corresponds to a density of 1/2 (which, as we will show, is now only true at the left boundary). However, maintaining the nomenclature is a reminder that the nature of the phases remains similar to those of TASEP, in the sense that they are still controlled by the in-rate (initiation rate *α*) for LD, the out-rate (termination rate *β*) for HD, and by the bulk hopping rate (elongation rate *k*) for MC.

## Results

### Density and current profiles

To derive the density profiles for the stochastic model of ribosome drop-off, we start by writing the master equations in the standard mean-field approximation, i.e., neglecting correlations between neighbouring lattice sites:

$$\frac{d\rho_{i}}{dt}=k\rho_{i-1}\left(1-\rho_{i}\right)-\gamma \rho_{i}-k\rho_{i}\left(1-\rho_{i+1}\right),$$ (5)

where we focus the discussion on the steady state, for which $\frac{d\rho_{i}}{dt}=0$.

Eq (5) gives access to the density profile, i.e the local density *ρ*_*i*_ for all sites *i* along the lattice. However, from a mathematical point of view it is more readily exploited in terms of a continuous function *ρ*(*x*) of a positional variable *x* which varies from 0 to *L* along the segment. Quite intuitively this amounts to defining the lattice spacing *ε* = *L*/*N*, where *N* is the number of sites in the lattice. We then require that *ρ*(*x*_*i*_) = *ρ*_*i*_ on all lattice points (for which *x*_*i*_ = *i ε*), and take the ‘continuous’ limit *ε* → 0 in order to make the lattice points densely cover the interval *x* ∈ [0, *L*]. A simple way of stating this is to say that, instead of the discrete lattice, we work with a much more refined one (and in fact an infinitely refined one). We already point out that this implies rescaling some variables [39] in order to make contact with the model predictions, as we will discuss below.

The continuous formulation is thus obtained by identifying *ρ*_*i*_ ≡ *ρ*(*x*), *ρ*_*i*+1_ ≡ *ρ*(*x* + *ε*), *ρ*_*i*−1_ ≡ *ρ*(*x* − *ε*), and by expanding the density *ρ*(*x*) in powers of *ε* to first order as

$$\rho \left(x\pm \varepsilon \right)\approx \rho \left(x\right)\pm \frac{\partial \rho (x)}{\partial (x)}\varepsilon .$$ (6)

For simplicity we will denote *ρ*(*x*)≡*ρ* and $\frac{\partial \rho (x)}{\partial (x)}\equiv \rho^{\prime }$ from now on, and omit the explicit reference to the spatial dependence.

We first establish the current-density relation *J*(*ρ*), by using Eq (6) to express the current *J*_*i*_ = *kρ*_*i*_(1 − *ρ*_*i*+1_) in the continuous limit:

$$J\left(x\right)=k\lim_{\varepsilon \to 0}\left[\rho \left(x\right)\;\left(1-\left(\rho \left(x\right)+\varepsilon \rho^{\prime }\left(x\right)\right)\right)\right]=k\;\rho \left(x\right)\left(1-\rho \left(x\right)\right).$$ (7)

This shows that, locally, the current still follows relation (4), valid for TASEP, and its variation along the strand is directly due to the density variation. In particular, this implies that the current achieves its maximum value at *ρ* = 1/2, and we shall see that a density *ρ*(0) = 1/2 at the entrance of the lattice still defines the MC phase. Moreover, due to relation (7), the LD and HD phases continue to be discriminated by the criterion *ρ*_*LD*_ < 1/2 and *ρ*_*HD*_ > 1/2, respectively. However, in these phases the current is no longer preserved along the lattice.

By substituting Eq (6) into Eq (5) we obtain a condition for the stationary state:

$$k\left(\rho -\rho^{\prime }\varepsilon \right)\left(1-\rho \right)-\gamma \rho -k\rho \left(1-\rho -\rho^{\prime }\varepsilon \right)=0.$$ (8)

Reordering terms yields

$$\rho^{\prime }=\frac{\gamma }{\varepsilon k}\frac{\rho }{2\rho -1},$$ (9)

At this point it becomes clear that, rather than by the individual parameters, the behaviour in the continuous limit is set only by the combination *γ*/*ε*. This suggests [39] defining a *rescaled drop-off rate* as

Γ=γ/ε. (10)

It is this variable which must be matched when comparing predictions to simulations on a discrete system (see also the Supplemental Material for an example). In the context of translation, we are dealing with *N* codons of a size of about 0.9 nm each, and therefore the rescaled drop-off rate to be used in the continuous model is related to the actual drop-off rate *γ* by Γ = *γ*/(0.9 nm).

Consequently, using the rescaled drop-off rate Γ = *γ*/*ε*, we thus establish a differential equation for the density profiles as:

$$\rho^{\prime }=\frac{\Gamma }{k}\frac{\rho }{2\rho -1}.$$ (11)

Notice that Eqs (7) and (11) imply

$$\frac{\partial J(\rho (x))}{\partial x}=\frac{\partial J(x)}{\partial \rho }\frac{\partial \rho (x)}{\partial x}=-\Gamma \rho \left(x\right),$$ (12)

which reflects our intuition about the drop-off process: the current decreases along the lattice due to particles dropping off at each site.

Eq 11 can be solved analytically by separating variables,

$$\left(2-\frac{1}{\rho }\right)d\rho =\frac{\Gamma }{k}dx.$$ (13)

Notice that the bounds to be applied are specific to the phase and the corresponding boundary conditions. For example, in the LD phase the density at *x* = 0 is given by *ρ*_*LD*_(0) = *α*/*k*, whereas in the HD phase the density at *x* = *L* is *ρ*_*HD*_(*L*) = 1 − *β*/*k*. Note that in the HD phase it is possible to maintain that value of the density at the right boundary despite the presence of drop-off, since the exit rate is more limiting than the drop-off rate, and therefore a queue of particles can still form. Essentially, all particles which drop off are replenished via the in-rate in this regime. However, there is a critical value of the drop-off rate beyond which the HD phase is not sustainable anymore, as we will see in the section ‘Phase Diagram’. Based on these boundary conditions we can then obtain the density profiles for the LD and the HD phases. Using *ρ*_*LD*_(0) = *α*/*k*, we get

$$\int_{\alpha /k}^{\rho_{LD}\left(x\right)}\left(2-\frac{1}{\rho }\right)d\rho =\int_{0}^{x}\frac{\Gamma }{k}d\tilde{x},$$ (14)

which leads to an implicit equation for the density profile:

$$\rho_{LD}\left(x\right)e^{-2\rho_{LD}\left(x\right)}=\frac{\alpha }{k}e^{-(\frac{\Gamma }{k}x+2\frac{\alpha }{k})}.$$ (15)

Similarly, by using *ρ*_*HD*_(*L*) = 1 − *β*/*k*, we get

$$\int_{\rho_{HD}\left(x\right)}^{\left(1-\beta /k\right)}\left(2-\frac{1}{\rho }\right)d\rho =\int_{x}^{L}\frac{\Gamma }{k}d\tilde{x},$$ (16)

and therefore:

$$\rho_{HD}\left(x\right)e^{-2\rho_{HD}\left(x\right)}=\left(1-\frac{\beta }{k}\right)e^{\frac{\Gamma }{k}\left(L-x\right)-2\left(1-\frac{\beta }{k}\right)}.$$ (17)

Finally, in the maximal current (MC) phase, we expect the current at the left boundary to be close to *k*/4, the TASEP value, since no significant amount of detachment can have occurred. Therefore, using *J*_*MC*_(0) = *k*/4 = *α*(1 − *ρ*(0)), the density should be $\rho_{MC}\left(0\right)=1-\frac{k}{4\alpha }$ at the left boundary. However, we cannot use *ρ*(0) as a bound for the integral in Eq (13): the continuous approach, in the above approximation, does not give access to the variation of the density profile within boundary layers. Instead we use the condition *J*_*MC*_(0) = *k*/4 = *kρ*(0)(1 − *ρ*(0)), which yields *ρ*_*MC*_(0) = 1/2. Using this bound for the integration, we obtain

$$\int_{1/2}^{\rho_{MC}\left(x\right)}\left(2-\frac{1}{\rho }\right)d\rho =\int_{0}^{x}\frac{\Gamma }{k}d\tilde{x},$$ (18)

which leads to

$$\rho_{MC}\left(x\right)e^{-2\rho_{MC}\left(x\right)}=\frac{1}{2}e^{-(\frac{\Gamma }{k}x-1)}.$$ (19)

Eqs (15), (17) and (19) for the density profiles *ρ*_*LD*_(*x*), *ρ*_*HD*_(*x*) and *ρ*_*MC*_(*x*) have an explicit solution in terms of the so-called Lambert function [38], denoted by *W* and defined as the inverse function of *y* = *f*(*z*) = *ze*^*z*^, i.e., *z* = *f*^−1^(*y*) = *W*(*y*). To see this one can multiply both sides of Eqs (15), (17) and (19) by −2, which yields an equation of the form *z*(*x*)*e*^*z*(*x*)^ = *y*(*x*), where *z*(*x*) = −2*ρ*(*x*) and *y*(*x*) stands for -2 times the right-hand side of Eqs (15), (17) and (19), respectively. Therefore the solution is given as *ρ*(*x*) = −*W*(*y*(*x*))/2.

**Fig 3:**
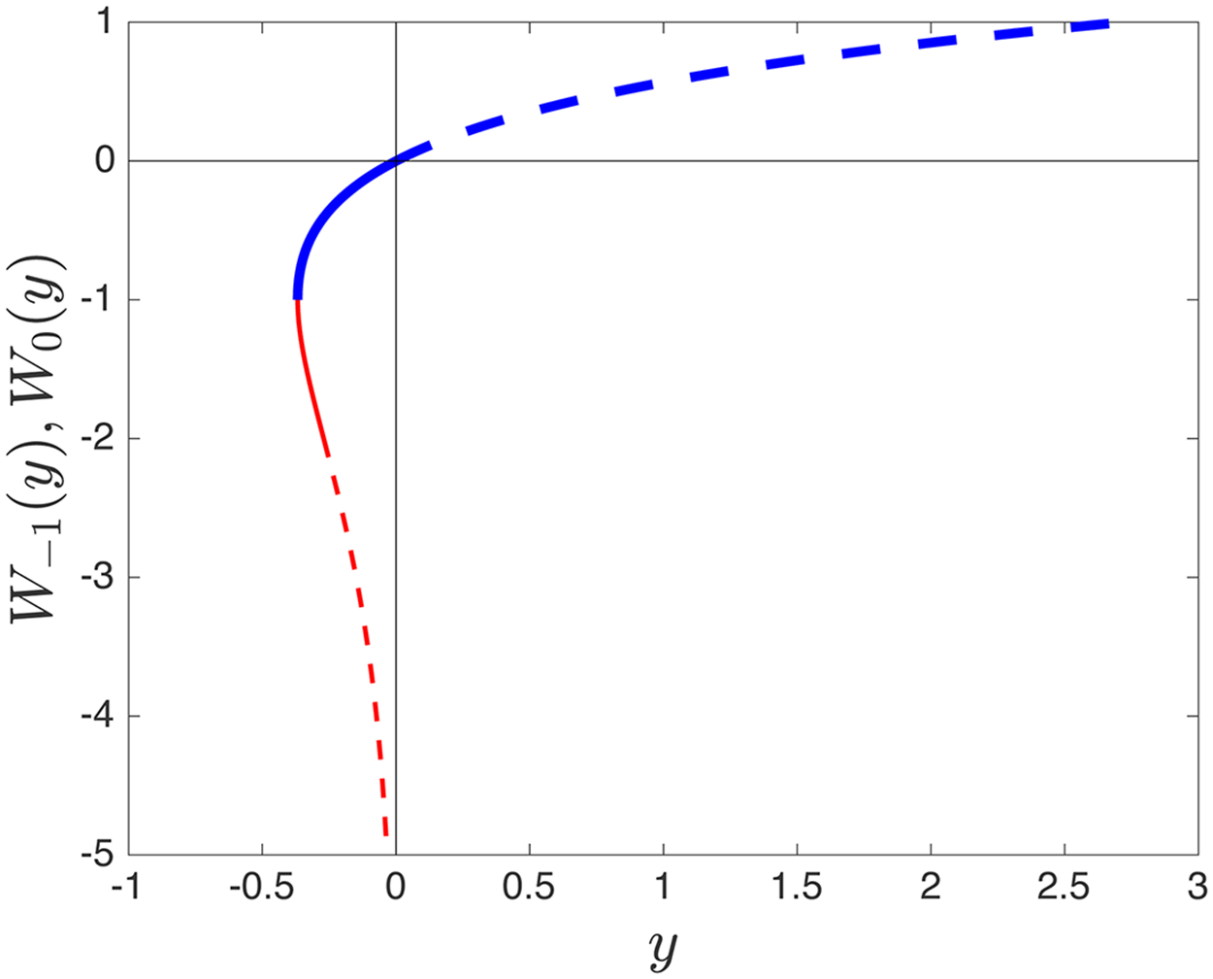
Both real branches *W*_0_(*y*) (bold blue line) and *W*_−1_(*y*) (red line) of the Lambert function. The solid part of the blue line corresponds to the low density and maximal current phases, and the solid part of the red line corresponds to the high density phase.

The *W* function is multivalued, involving two main branches denoted as *W*_0_ and *W*_−1_ (see Fig 3). Hence, for the equations above to be well defined we need to identify the appropriate branch of the Lambert function in each case, i.e. for the LD, HD and MC phases. To do this we consider the following: since in the LD and MC phases the particle density on the lattice is between 0 and 0.5, the branch of interest in these two cases is *W*_0_(*y*) (blue thick line), and specifically the region marked with the solid blue line in Fig 3. Analogously, since the particle density in the HD phase is between 0.5 and 1, the branch of interest is *W*_−1_(*y*) (red line), in the region where it highlighted as the solid red line in Fig 3.

Hence, an analytical expression can be given for the density profile in the LD regime:

$$\rho_{LD}\left(x\right)=-\frac{1}{2}W_{0}\left(y_{LD}\left(x\right)\right),$$ (20)

where

$$y_{LD}\left(x\right)=-2\frac{\alpha }{k}e^{-\frac{\Gamma }{k}x-2\frac{\alpha }{k}}.$$ (21)

The HD phase can be discussed analogously. The density profile is given by

$$\rho_{HD}\left(x\right)=-\frac{1}{2}W_{-1}\left(y_{HD}\left(x\right)\right),$$ (22)

where

$$y_{HD}\left(x\right)=-2\;\left(1-\frac{\beta }{k}\right)e^{\frac{\Gamma }{k}\left(L-x\right)-2\left(1-\frac{\beta }{k}\right)}.$$ (23)

And, finally, in the MC phase the density profile is given by

$$\rho_{MC}\left(x\right)=-\frac{1}{2}W_{0}\left(y_{MC}\left(x\right)\right),$$ (24)

where

$$y_{MC}\left(x\right)=-e^{-\frac{\Gamma }{k}x-1}.$$ (25)

The expressions for *ρ*_*MC*_, *ρ*_*LD*_ and *ρ*_*HD*_ depend on the ratio Γ/*k*, as well as on *α*/*k* and *β*/*k* in the LD and HD cases, respectively. We thus define the rescaled parameters $\tilde{\Gamma }=\Gamma /k$, $\tilde{\alpha }=\alpha /k$ and $\tilde{\beta }=\beta /k$ and use them instead of the original parameters from now on.

**Fig 4:**
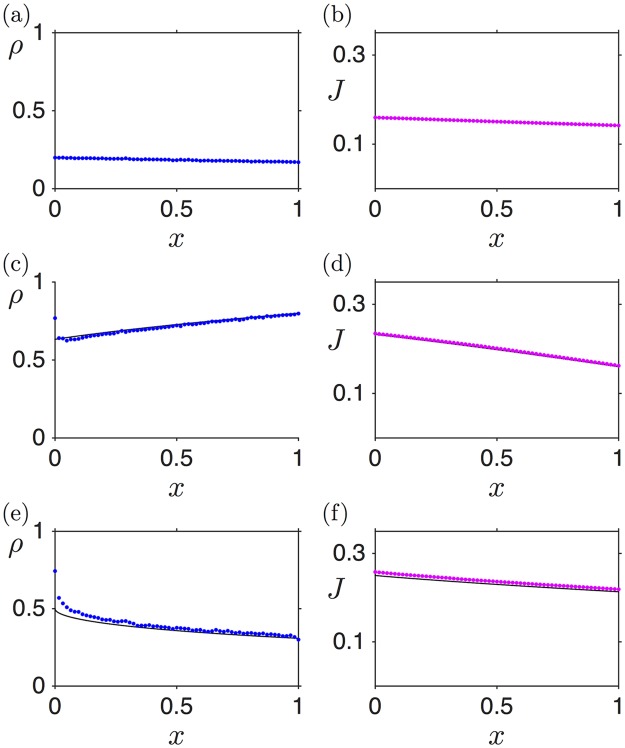
Density and current profiles numerically computed via stochastic simulations (blue and magenta dots, respectively) and analytically estimated (black lines) for $\tilde{\Gamma }=0.1$ and *L* = 1: (a, b) LD: $\tilde{\alpha }$ = 0.2, $\tilde{\beta }$ = 1; (c, d) HD: $\tilde{\alpha }$ = 1, $\tilde{\beta }$ = 0.2; (e, f) MC: $\tilde{\alpha }$ = 1, $\tilde{\beta }$ = 1. Hopping and drop-off events are scheduled based on the Gillespie algorithm [41]. All simulations, here and in the following, are for 10^7^ iteration steps (each iteration corresponds to one reaction: initiation, elongation, drop-off or termination). The first 2 ⋅ 10^7^ iterations were discarded to make sure that the system was in a steady state. Unless otherwise stated, a lattice size of 500 sites was used.

Fig 4 shows three density profiles, alongside the corresponding current profiles, for the LD (a,b), HD (c,d) and MC (e,f) phases. The analytical expressions obtained above (black lines) show good agreement with the numerical simulations (blue points), apart from the boundary layers. Note that this disagreement at the boundaries decreases with increasing number of lattice sites [39].

In the LD case, the density profile decreases from left to right (Fig 4(a)). This is as expected: the entry rate fixes the density at the left boundary, and due to the drop-off rate some particles are lost on their journey to the 3’ end (right-hand boundary) of the lattice, causing a decreasing density profile. For the HD phase, however, the ribosome density profile counter-intuitively increases from left to right (5’ to 3’ end) (Fig 4(c)). One could expect that, since more and more ribosomes drop off as they travel along the mRNA, their density should *always decrease* along the strand. However, in the HD phase there is another factor: as drop-off progressively depletes the ribosome population, their motion is less impeded, which increases the current with respect to the case without ribosome drop-off (see Fig 4(d); the corresponding current without ribosome drop-off would be equal to *J*(*L*) and constant throughout the mRNA (not shown)). This constitutes a compensatory mechanism, which ultimately leads to a build-up of particles downstream the segment, i.e. an *increasing* density profile. It is the balance between these two facts which sets the density slope. Mathematically, this can be seen by considering Eq (12), which implies that *J*(*x*) is a non-increasing function of *x*. Moreover, *J*(*x*) is a symmetric function of *ρ* with a maximum at *ρ* = 1/2. Therefore, if *ρ*(0) > 1/2, like in HD, then *ρ*(*x*) must be increasing to allow *J*(*x*) to decrease. Analogously, if *ρ*(0) < 1/2, like in LD, *ρ*(*x*) must be decreasing (we thank the anonymous reviewer for providing this argument).

A further important characteristic of mRNAs sustaining an HD phase is that, also somewhat counter-intuitively, ribosome drop-off induces an increase in the current at the 5’ end of the mRNA. This is simply due to the fact that the HD phase is limited by the termination rate: therefore current and density at the 3’ end are directly set by the termination rate, at the TASEP values. However, the current decreases along the strand, as discussed above, implying that the current lies above the TASEP value anywhere else in the segment. Summarising, on mRNAs carrying a HD phase, ribosome drop-off induces a current increase at the 5’ end.

### Localised domain wall

Much useful insight into the phase diagram can be gained from understanding the circumstances under which a shock phase arises, i.e. when different phases co-exist in upstream and downstream zones. In the presence of drop-off, the position of this domain wall is localised, i.e. the associated density mismatch arises at a specific position along the lattice [39]. This is in contrast to what is seen for the regular TASEP, and we first recall the domain wall phenomenology in this latter case.

In the conventional TASEP, without drop-off, the shock phase (SP) arises between an LD (upstream) zone and a HD (downstream) zone, when *α* = *β*. However, the position of the domain wall will fluctuate randomly [45], as is most easily seen by considering the case of very small input/output rates, *α* = *β* ≪ 1. In this regime the entry/exit of particles are rare events, and the densities on the respective sections of the lattice are *ρ*_*LD*_ ≃ 0 and *ρ*_*HD*_ ≃ 1 (see ‘Materials and Methods’). We are thus essentially dealing with a queue of particles in front of the exit site, extending up to the domain wall position *x*_*w*_. Note that this is a valid stationary state, despite the different densities at different sides of the domain wall, since both zones carry the same current (see *J*_*LD*_ and *J*_*HD*_ in ‘Materials and Methods’ for *α* = *β*). Consider now an isolated event of an additional particle entering the lattice. Since the exit rate is also very small, the most likely scenario is that the extra particle can reach the domain wall, where it gets stuck. This effectively moves the domain wall upstream by approximately one lattice site, at rate *α*. Alternatively, the event of a particle leaving the lattice will displace the domain wall downstream by (approximately) one lattice site, at rate *β*. In both cases the new domain wall position corresponds again to a stationary state, since all currents remain unaltered. Since both domain-wall displacements occur with the same rate (*α* = *β*), the domain wall is seen to perform a random walk, and therefore to diffuse freely all over the lattice. This argument can be easily extended to the case of general *α* = *β* < 1/2, i.e. it is general to the shock phase in TASEP, and its dynamics are well understood [45].

The presence of drop-off introduces an important difference: the density profiles, and therefore the current profiles, are now position-dependent even throughout the bulk of the LD and HD zones. Nevertheless, the current still has to be continuous across the domain wall: otherwise, the mismatch of currents would locally redistribute particles across the domain wall, which would thereby be displaced until the currents match. Therefore, there is one particular position *x*_*w*_ at which the domain wall can remain, and it is set through the condition *J*_*LD*_(*x*_*w*_) = *J*_*HD*_(*x*_*w*_), which leads to

$$\rho_{LD}\left(x_{w}\right)=1-\rho_{HD}\left(x_{w}\right).$$ (26)

**Fig 5:**
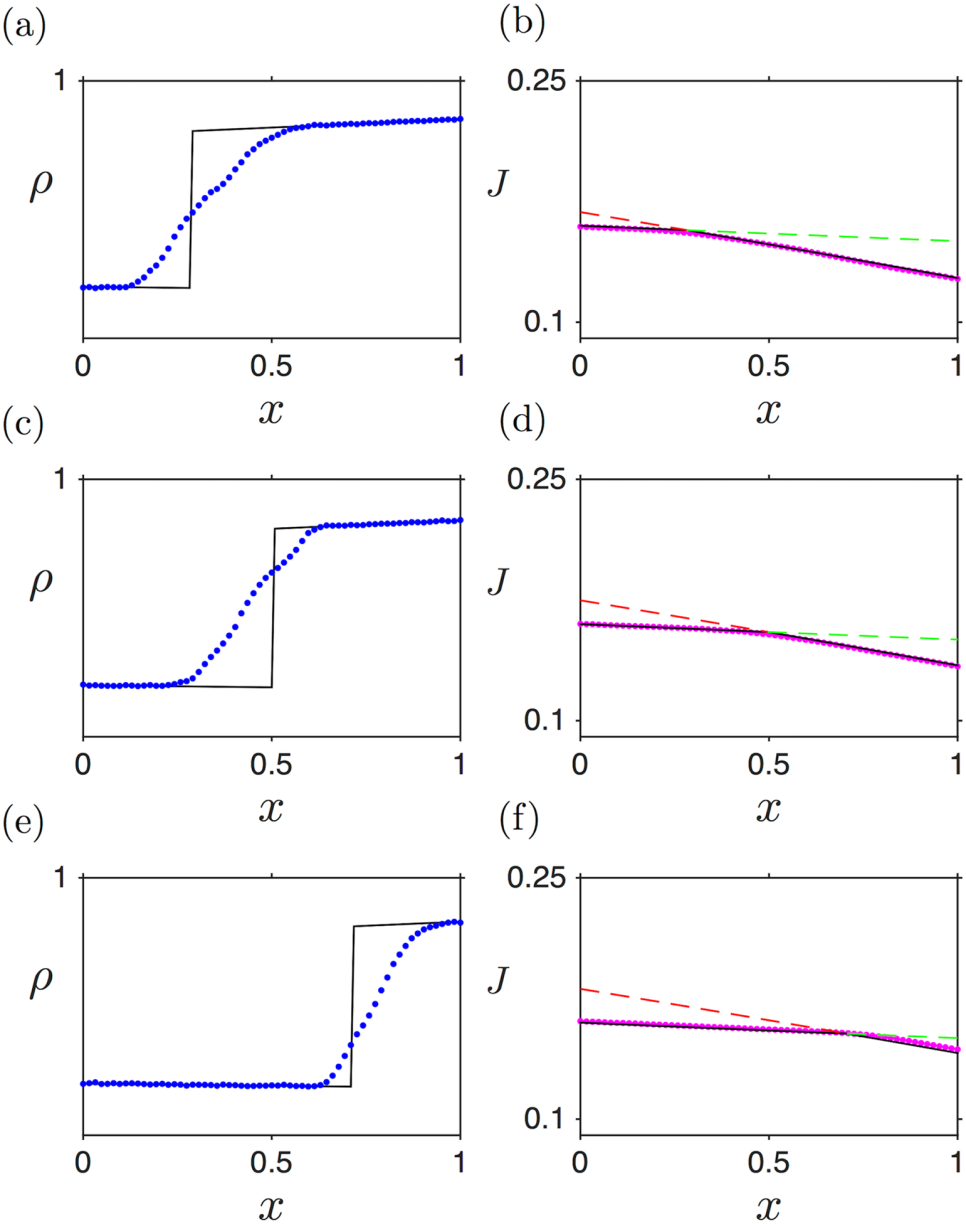
Density and current profiles in the shock phase, for $\tilde{\Gamma }=0.05$, *L* = 1 and $\tilde{\alpha }=0.2$. (a,b) $\tilde{\beta }=0.15$, (c,d) $\tilde{\beta }=0.16$ and (e,f) $\tilde{\beta }=0.17$. Numerical simulations are shown as blue (density) and magenta (current) points, whereas analytical predictions for the density profiles are shown as black lines. For the current profiles the analytical expressions for *J*_*LD*_(*x*) and *J*_*HD*_(*x*) are shown with green and red dashed lines, respectively.

This is also seen directly from the dynamic picture given above. Consider an entrance event, so that the domain wall would be pushed upstream, say to *x*_*w*_ − *ϵ*. As is apparent from the current profile, e.g. in Fig 5(f), at this new position the current corresponding to the HD segment is higher than the current of the LD segment. The resulting imbalance therefore leads to the domain wall moving downstream, restoring the initial domain wall position. Using the analogous argument for an exit event, it becomes clear that there is indeed a privileged position at which the domain wall can remain stationary. It is easy to generalise the argument to arbitrary values of *α* and *β*, but one may wonder whether the stabilisation mechanism always applies. Indeed, it requires the current slope of the LD phase to be weaker, at the position of the domain wall, than that of the HD phase. But this condition is seen to be always fulfilled by using Eq (12) as well as the fact that *ρ*_*LD*_(*x*) < *ρ*_*HD*_(*x*) anywhere at the lattice.

A closer analysis shows another new feature due to drop-off: there is now a second type of shock, which involves coexistence between the HD and MC phases. Indeed, as we discussed above, in the HD phase the loss of ribosomes along the mRNA leads to a ribosome density profile which increases from 5’ to 3’. In the MC phase, on the other hand, it decreases. Just as in the case of LD-HD coexistence, it is possible to have MC-HD coexistence separated by a domain wall, located at *x*_*w*_, such that the current remains continuous, i.e. *J*_*MC*_(*x*_*w*_) = *J*_*HD*_(*x*_*w*_). This condition leads to

$$\rho_{MC}\left(x_{w}\right)=1-\rho_{HD}\left(x_{w}\right).$$ (27)

Importantly, there is a density mismatch at the domain wall position, just as for the LD-HD coexistence phase, and we are thus again dealing with a proper coexistence associated with the MC-HD transition. Note that this contrasts the regular TASEP, where this transition is continuous. Note also that there is no further type of coexistence: using Eqs (20, 21) and (24, 25), together with Eq (7) one can see that the currents in the LD and MC phases coincide only if $\tilde{\alpha }=1/2$. At this point the LD and MC phases have identical density profiles, which excludes the scenario of a domain wall and therefore implies a second order transition.

In the following we refer to a phase featuring a domain wall as a ‘shock phase’ (SP), and we only distinguish between a LD-HD and a MC-HD shock phase when this is useful. According to the nature of the interface, either Eq (26) or Eq (27) must hold across the domain wall due to current conservation, from which a unique domain wall position *x*_*w*_ follows. It will be useful in the following to realise that the value of *x*_*w*_ indirectly indicates how the shock phase evolves as one of the rates (initiation, elongation or termination) is varied. Indeed, for a shock to be present the domain wall must lie within the bulk of the lattice (*x*_*w*_ ∈ [0, *L*]). However, a domain wall position reducing to zero (*x*_*w*_ ≤ 0) indicates that the domain wall has moved to the left boundary, which implies that the HD phase is established all along the lattice. Analogously, *x*_*w*_ ≥ *L* means that the domain wall has moved to the very right boundary, and therefore the LD phase is established across the entire lattice.

**Fig 6:**
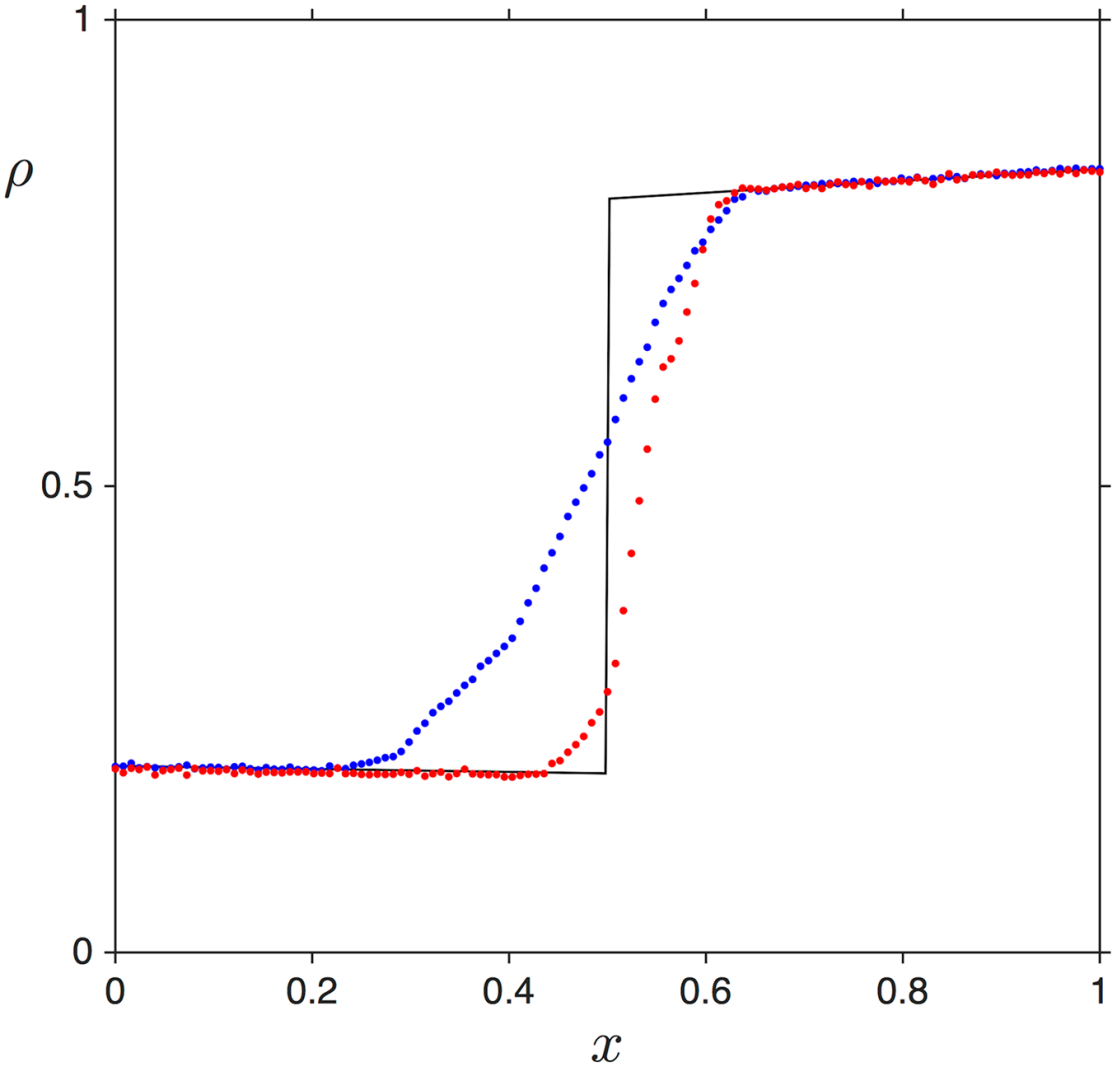
Illustration of finite size effects in the shock phase, based on the density profile for $\tilde{\Gamma }=0.05$, $\tilde{\alpha }=0.2$, $\tilde{\beta }=0.16$ and *L* = 1. The blue dots represent the numerical results for *N* = 500 sites,whereas red dots are for *N* = 1000 sites. The solid black line corresponds to the analytical solution.

We illustrate this in Fig 5, which shows three density and current profiles corresponding to the SP, for identical conditions except for the exit rate $\tilde{\beta }$ which is progressively increased (0.15 in panels a,b; 0.16 in panels c,d; and 0.17 in panels e,f). We see how the position of the shock evolves towards the right boundary (3’ end) as the value of $\tilde{\beta }$ increases. Eventually, when $\tilde{\beta }$ becomes small enough, the domain wall attains *x*_*w*_ = 0, which indicates that the high density domain occupies the entire lattice beyond this threshold. Predictions from the analytical expressions show good agreement with simulation data, apart from the transition zone from low to high density, close to the position of the domain wall. This discrepancy can be attributed to the fact that finite size effects are present: indeed, Fig 6 shows how the transition becomes sharper as we increase the system size.

### Phase diagram

Having established a complete catalogue of the phases, we can now construct the phase diagram, and comment in particular on those phases where shocks appear. The full phase diagram must specify, for each point $(\tilde{\alpha },\tilde{\beta }$) in the phase plane, the phase corresponding to these parameters.

The most straightforward observation to make is that the line separating the LD and MC phase remains unchanged with respect to TASEP, i.e. it occurs at $\tilde{\alpha }=1/2$. This can be seen from Eqs (20, 21) and (24, 25), having in mind that the density in LD must respect the condition *ρ*_*LD*_ < 1/2. As we have seen, the LD density profile decreases along the sequence in the presence of drop-off, and therefore this condition will first be violated at the left boundary (*x* = 0) as the rate $\tilde{\alpha }$ is progressively increased. We can thus evaluate the boundary of the LD phase through the criterion *ρ*_*LD*_(0) = 1/2, which yields $\tilde{\alpha }=1/2$. Recall that this transition from LD to MC is continuous, as already discussed above: this is seen here directly by substituting $\tilde{\alpha }=1/2$ into Eq (20).

As a second element to the phase diagram, we recall that the ‘shock phase’ (SP) now englobes two different kinds of coexistence, LD-HD (for $\tilde{\alpha }<1/2$) and MC-HD (for $\tilde{\alpha }>1/2$). They too are therefore separated by a line at $\tilde{\alpha }=1/2$.

The remainder of the phase diagram can now conveniently be established by determining the boundaries of the SP, i.e. by determining the conditions at which coexistence ceases. As discussed in the previous section, the SP can be identified as those points $\left(\tilde{\alpha },\tilde{\beta }\right)$ for which either Eq (26) or Eq (27) is fulfilled, recalling that *x*_*w*_ must lie within the interval [0, 1]. Equating *x*_*w*_ = 0 amounts to calculating the transition from HD to SP (since the HD has taken over the entire mRNA lattice when the domain wall is located at the left boundary). Analogously, equating *x*_*w*_ = *L* corresponds to calculating the SP to LD transition. Here we can invoke our insight established above and illustrated in Fig 5: at a fixed $\tilde{\alpha }$ we expect the domain wall to be pushed towards the entrance (5’ end) as $\tilde{\beta }$ is reduced, and towards the exit (3’ end) as $\tilde{\beta }$ is increased. Therefore there are both a lower bound ${\tilde{\beta }}_{l}\left(\tilde{\alpha }\right)$ and an upper bound ${\tilde{\beta }}_{u}\left(\tilde{\alpha }\right)$ to the SP zone. We can determine these by solving for *x*_*w*_ = 0 and *x*_*w*_ = *L*, respectively, distinguishing the cases $\tilde{\alpha }<1/2$ (for which the SP coexistence is LD-HD), and $\tilde{\alpha }>1/2$ (for which it will be LD-MC).

Specifically, the upper bound of the SP can be determined by setting *x*_*w*_ = *L* in Eq (26) for $\tilde{\alpha }\leq 1/2$, and in Eq (27) for $\tilde{\alpha }\geq 1/2$, and then solving for $\tilde{\beta }$ as a function of $\tilde{\alpha }$. This yields

$${\tilde{\beta }}_{u}=\left\{\begin{array}{cc} -\frac{1}{2}W_{0}\left(-2\tilde{\alpha }e^{-2\tilde{\alpha }-\tilde{\Gamma }L}\right), & \text{if}\;0<\tilde{\alpha }\leq 1/2,\\ -\frac{1}{2}W_{0}\left(-e^{-1-\tilde{\Gamma }L}\right), & \text{if}\;\tilde{\alpha }>1/2, \end{array}\right.$$ (28)

which defines the LD-SP and MC-SP phase boundaries, respectively. Notice that the transition between both boundaries is continuous at $\tilde{\alpha }=1/2$, as expected.

In the same manner, the SP-HD boundary is determined by setting *x*_*w*_ = 0 in Eq (26) for $\tilde{\alpha }\leq 1/2$, and in Eq (27) for $\tilde{\alpha }\geq 1/2$, and solving for $\tilde{\beta }$ as a function of $\tilde{\alpha }$:

$${\tilde{\beta }}_{l}=\left\{\begin{array}{cc} 1+\frac{1}{2}W_{-1}\left(-2\left(1-\tilde{\alpha }\right)e^{-2\left(1-\tilde{\alpha }\right)-\tilde{\Gamma }L}\right) & \text{if}\;0<\tilde{\alpha }\leq 1/2\\ 1+\frac{1}{2}W_{-1}\left(-e^{-1-\tilde{\Gamma }L}\right) & \text{if}\;\tilde{\alpha }>1/2, \end{array}\right.$$ (29)

which again yields a continuous transition at $\tilde{\alpha }=1/2$.

The conditions for the different phases can therefore be summarised as follows:

- LD phase: $0<\tilde{\alpha }\leq 1/2$ and $\tilde{\beta }>{\tilde{\beta }}_{u}$.
- MC phase: $\tilde{\alpha }\geq 1/2$ and $\tilde{\beta }>{\tilde{\beta }}_{u}$.
- HD phase: $\tilde{\beta }<{\tilde{\beta }}_{l}$.
- SP phase: ${\tilde{\beta }}_{l}<\tilde{\beta }<{\tilde{\beta }}_{u}.$

Alternatively, the maximum current principle [44] can be invoked to deduce the LD-MC and HD-MC boundaries. The maximum current principle states that the value of the current in the MC phase is given by *J*_*MC*_ = max_[*ρ*_*N*+1_, *ρ*_0_]_ *J*(*ρ*), where *ρ*_0_ and *ρ*_*N*+1_ represent densities associated to particles reservoirs on the left and right hand side of the lattice, respectively. Since in our ribosome drop-off model the fundamental relationship *J*(*ρ*) = *kρ*(1 − *ρ*) remains valid, the maximum in the current also occurs at *ρ* = 1/2. Then, by realising that the maximum value of *ρ* in LD can only be reached at *x* = 0, and equating *ρ*_*LD*_(0) = 1/2 we obtain the value of $\tilde{\alpha }$ at which the LD-MC transition occurs, namely $\tilde{\alpha }=1/2$. Analogously, by realising that the value of *ρ* that maximises the current in HD can only occur at *x* = 0, one can equate *ρ*_*HD*_(0) = 1/2, which yields $\tilde{\beta }=1+\frac{1}{2}W_{-1}\left(-e^{-1-\tilde{\Gamma }L}\right)$. Note that these values for where the boundaries occur are consistent with the ones obtained by the previous approach.

**Fig 7:**
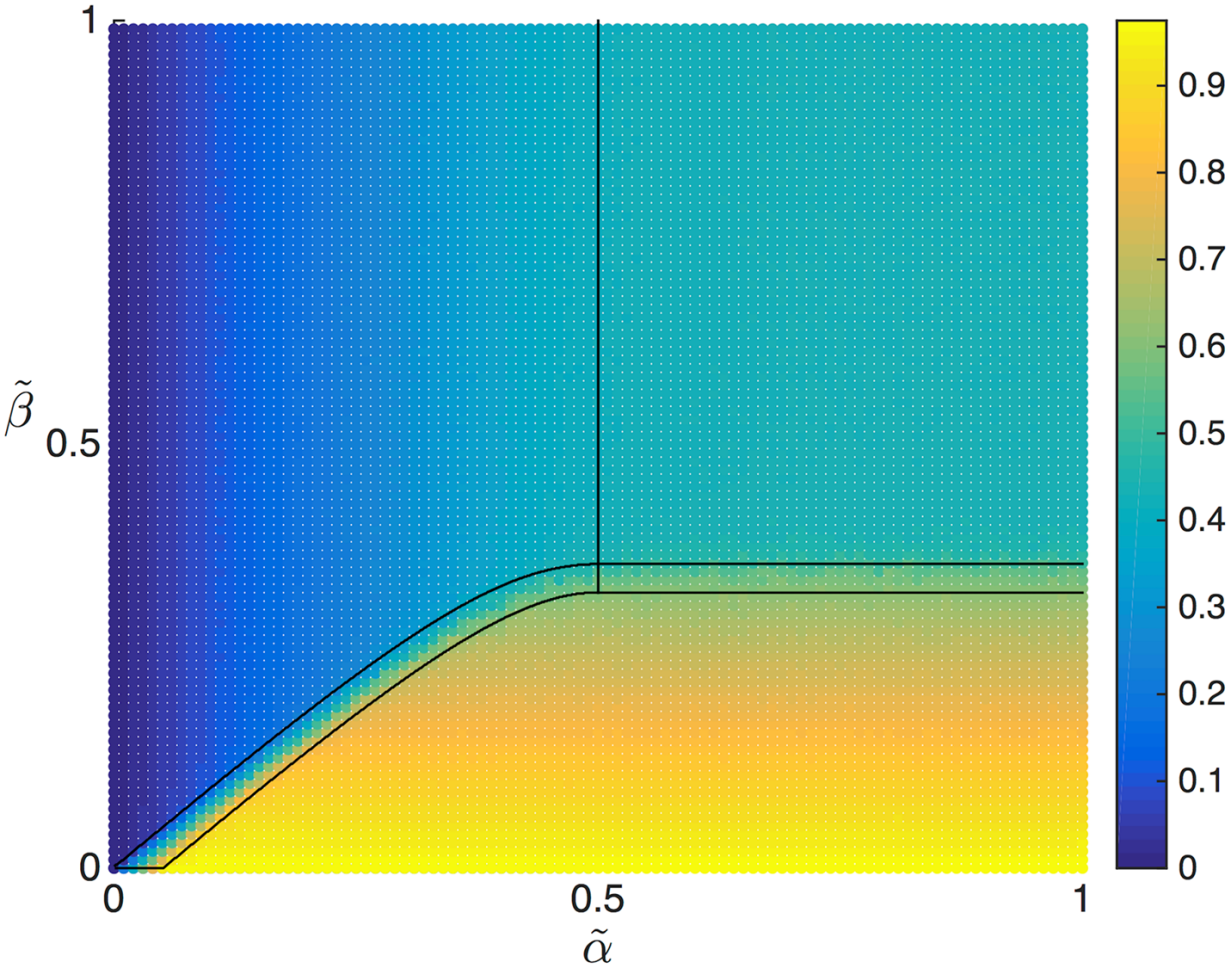
Phase diagram for $\tilde{\Gamma }=0.05$ and *L* = 1 showing the average density on the lattice (heat map) numerically computed via stochastic simulations, and the borders among the phases (black lines) estimated analytically within the mean-field approximation.

**Fig 8:**
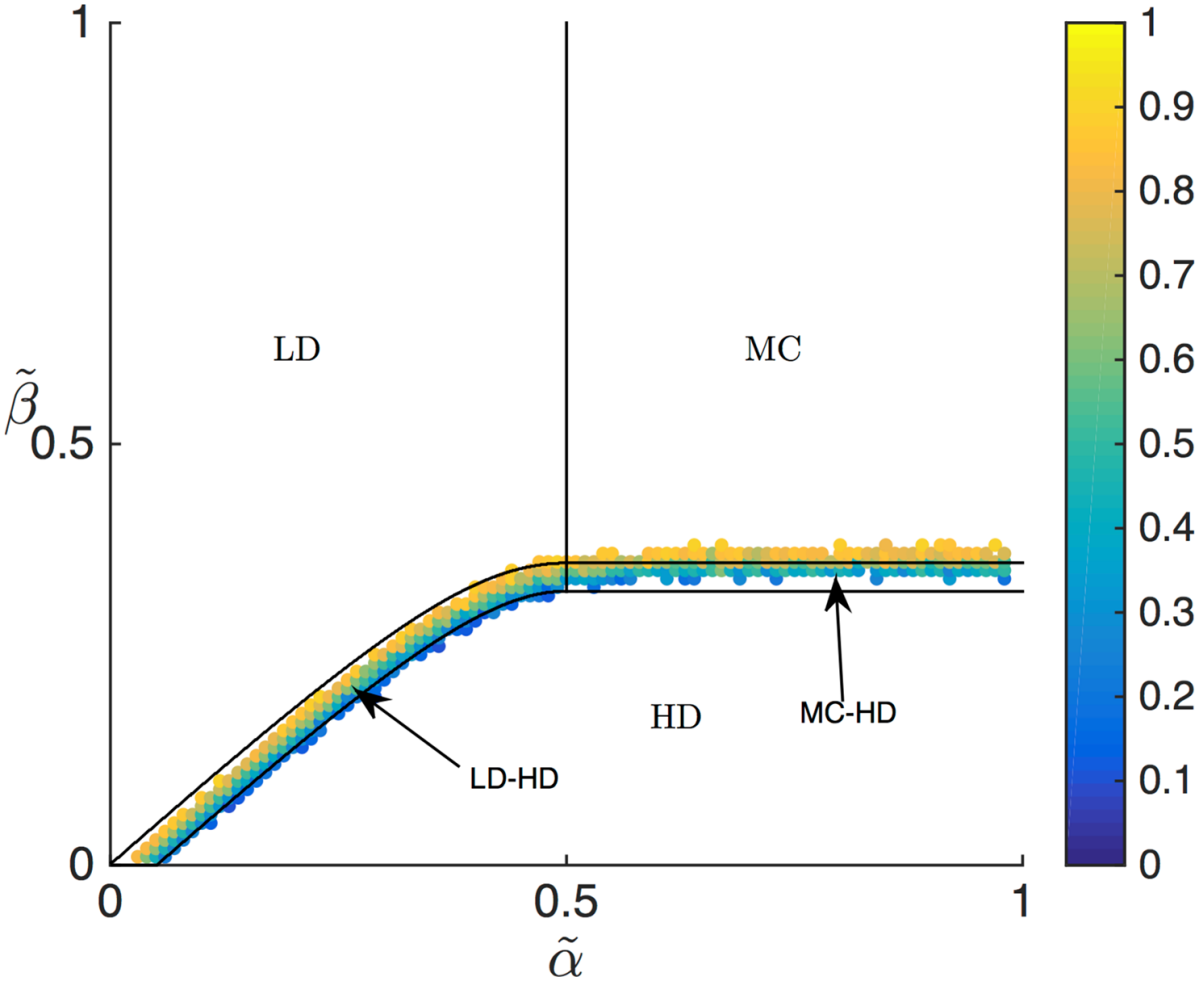
SP phase in the phase diagram, for $\tilde{\Gamma }=0.05$ and *L* = 1. The colour map represents the value of *x*_*w*_ for the position of the domain wall between LD and HD zones, as determined numerically. Where no data is shown *x*_*w*_ was negative or larger than *L*, indicating respectively a LD or a HD state point. The represented points thus identify the zone corresponding to a SP, which is to be confronted to the analytical estimation of its phase boundaries (black lines).

In order to validate this analysis we have used simulation data to calculate numerically the average density on the lattice, sampling points ($\tilde{\alpha },\tilde{\beta })$ across the phase diagram. This average density is shown, together with the phase boundaries obtained from the mean-field approach, in Fig 7. As expected, the average density calculated from simulation clearly distinguishes between the LD, HD and MC phases (the latter showing a constant average density across the entire phase, also as expected). Furthermore, to numerically validate the presence of a domain wall we have calculated the value at which the density *ρ*(*x*) crosses 0.5 from below (after applying a moving average filter to the simulated density profile spanning 50 codons): this indicates a domain wall, and cannot happen in a LD, HD or MC phase. This method thus provides a straightforward tool to numerically determine the SP region, and furthermore to determine the position of the domain wall. Fig 8 shows those points ($\tilde{\alpha },\tilde{\beta })$ for which a domain wall has been detected from simulation data. The corresponding zone coincides reasonably well with the mean-field prediction for the SP region in the phase diagram, with a slight disagreement, most clearly present for $\tilde{\alpha }>1/2$.

**Fig 9:**
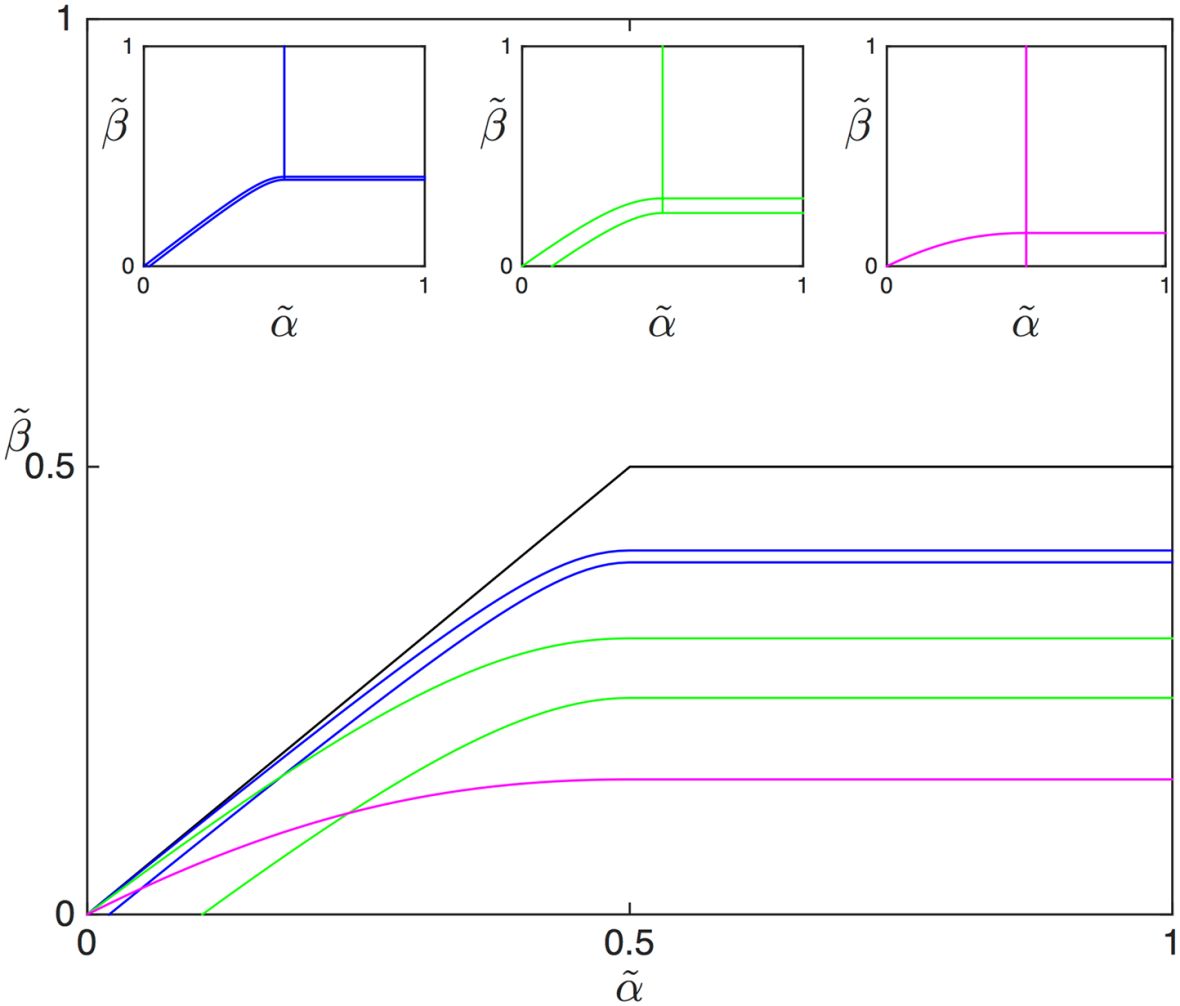
Extent of the MC-HD shock phase for *L* = 1, depending on $\tilde{\Gamma }$ (analytical results); black line: $\tilde{\Gamma }=0$, blue lines: $\tilde{\Gamma }=0.01$, green lines: $\tilde{\Gamma }=0.1$ and the purple line: $\tilde{\Gamma }=0.5$. The insets show the full phase diagram for these three values.

To complement the previous analysis, for which a particular value of $\tilde{\Gamma }$ was chosen, we now show how the extent of the SP region depends on $\tilde{\Gamma }$. To this end Fig 9 represents the boundaries of the SP region for various values of $\tilde{\Gamma }$, superposed using different colours. The TASEP case without drop-off ($\tilde{\Gamma }=0$), for which the SP zone collapses to a line, is shown as a reference (black line). Initially, as $\tilde{\Gamma }$ increases, the SP region widens and is progressively shifted to lower values of *β* (blue and green lines). During this process the LD, MC and SP regions expand at the expense of the HD region. The HD zone ultimately vanishes completely as the lower line delimiting the SP zone is shifted downwards, out of the represented portion of the $\left(\tilde{\alpha },\tilde{\beta }\right)$ plane.

**Fig 10:**
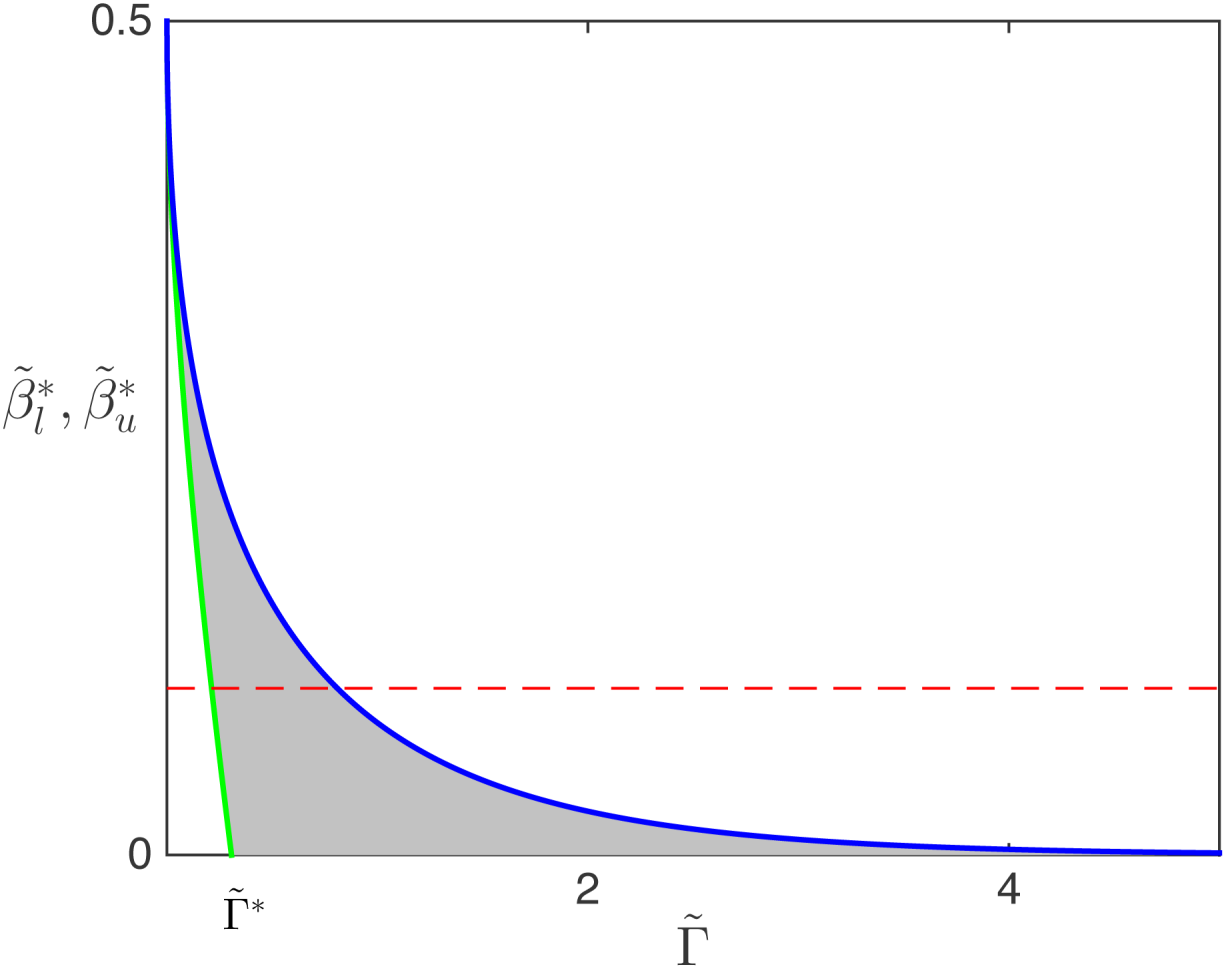
Analytically computed upper and lower boundaries to the MC-HD shock phase, depending on $\tilde{\Gamma }$ for *L* = 1. The green line represents the lower boundary ${\tilde{\beta }}_{l}^{*}$ and the blue line corresponds to the upper boundary ${\tilde{\beta }}_{u}^{*}$. The horizontal red dashed line serve as illustration. The intersections (at $\tilde{\beta }=0.1$) with ${\tilde{\beta }}_{l}^{*}$ and ${\tilde{\beta }}_{u}^{*}$ indicate the values of the drop-off rates at which an mRNA with termination rate $\tilde{\beta }=0.1$ would undergo a transition from HD to SP, and from SP to MC, respectively.

A complementary view of this progression is shown in Fig 10, where we quantify the extent of the SP zone which involves an MC-HD interface ($\tilde{\alpha }>1/2$) through its upper bound (blue line, ${\tilde{\beta }}_{u}^{*}=\beta_{u}$) and its lower bound (green line, ${\tilde{\beta }}_{l}^{*}=\max\left(0,{\tilde{\beta }}_{l}\right)$) as a function of $\tilde{\Gamma }$. In the absence of drop-off ($\tilde{\Gamma }=0$) both bounds are equal to 0.5, reproducing the behaviour known for TASEP. Then, as $\tilde{\Gamma }$ increases, the zone between ${\tilde{\beta }}_{l}$ and ${\tilde{\beta }}_{u}$ drops and widens, until ${\tilde{\beta }}_{l}^{*}=0$ reaches zero: this point represents the ribosome drop-off rate ${\tilde{\Gamma }}^{*}$ at which the HD phase becomes entirely unsustainable on the lattice. Using Eq (29) this value can be calculated as

$${\tilde{\Gamma }}^{*}=\frac{1-\ln2}{L},$$ (30)

indicated in Fig 10. Once the threshold ${\tilde{\Gamma }}^{*}$ has been crossed, the SP is delimited by the condition $0<\tilde{\beta }<{\tilde{\beta }}_{u}^{*}$. However, since the upper boundary ${\tilde{\beta }}_{u}^{*}$ of the SP asymptotically tends to 0 as $\tilde{\Gamma }\to \infty$, the extent of the SP region progressively vanishes for $\tilde{\Gamma }\to \infty$, with the LD and MC regions taking over the phase diagram: both HD and coexistence phases become inviable if the drop-off rate is too high.

### Ribosome drop-off effects depend strongly on codon configuration

The phase diagram is not only key to a systematic characterisation of the model, but is furthermore central to understand biological implications. The link is provided by a genome-wide analysis of ribosome traffic dynamics in *S. cerevisiae* [17], which has shown that different mRNAs can be classified according to the type of phase transition they present as the initiation rate is increased: in general, mRNAs can either undergo an LD-HD-like transition, or an LD-MC-like transition. Importantly, this classification has also shown a statistically significant correlation with the biological function encoded by the mRNA. For example, mRNAs encoding protein components of the ribosome and translational machinery predominantly undergo an LD-MC-like phase transition as the initiation rate *α* is increased. In contrast, mRNAs coding for regulatory proteins, such as transcription factors, predominantly undergo an LD-HD-like transition with increasing *α*. It was found that GO annotations (process) related to stress responses were significantly over-represented in mRNA sequences undergoing an LD-HD-like transition: biological regulation (P-value 1.74 × 10^11^) and cellular response to stimulus (P-value 1.79 × 10^−8^). Moreover, GO annotations (process) related to translation were significantly over-represented in mRNAs undergoing LD-MC-like transitions: cytoplasmic translation (P-value 7.04 × 10^−14^), translation (P-value 8.58 × 10^−8^). The P-value was computed using the hypergeometric distribution, and the Bonferroni correction was applied to take into account multiple hypothesis [54].

This prompts the question whether mRNAs in different phases are affected differently by the ribosome drop-off effect. If they react differently to drop-off, then this could indicate a further regulatory mechanism. In order to quantify the impact of ribosome drop-off, we consider the ribosome current at position *x* = *L* of the lattice, since this is the quantity that corresponds to the protein synthesis rate (full-length proteins produced per unit time). In particular, we consider the ratio

$$\chi_{\tilde{\Gamma }}=\frac{J_{\tilde{\Gamma }}\left(L\right)}{J_{0}\left(L\right)},$$ (31)

where *J*_0_(*L*) denotes the protein translation rate of that same mRNA sequence in the absence of ribosome drop-off. We refer to $\chi_{\tilde{\Gamma }}$ as the ribosome drop-off resilience of a given mRNA, since $0<\chi_{\tilde{\Gamma }}\leq 1$ characterises the extent to which the tranlation current is maintained despite the drop-off, where all other rates are maintained constant. By using Eqs (20), (22) and (24), together with the expression for the current Eq (7), we can then calculate the drop-off resilience in all LD, HD and MC phases.

**Fig 11:**
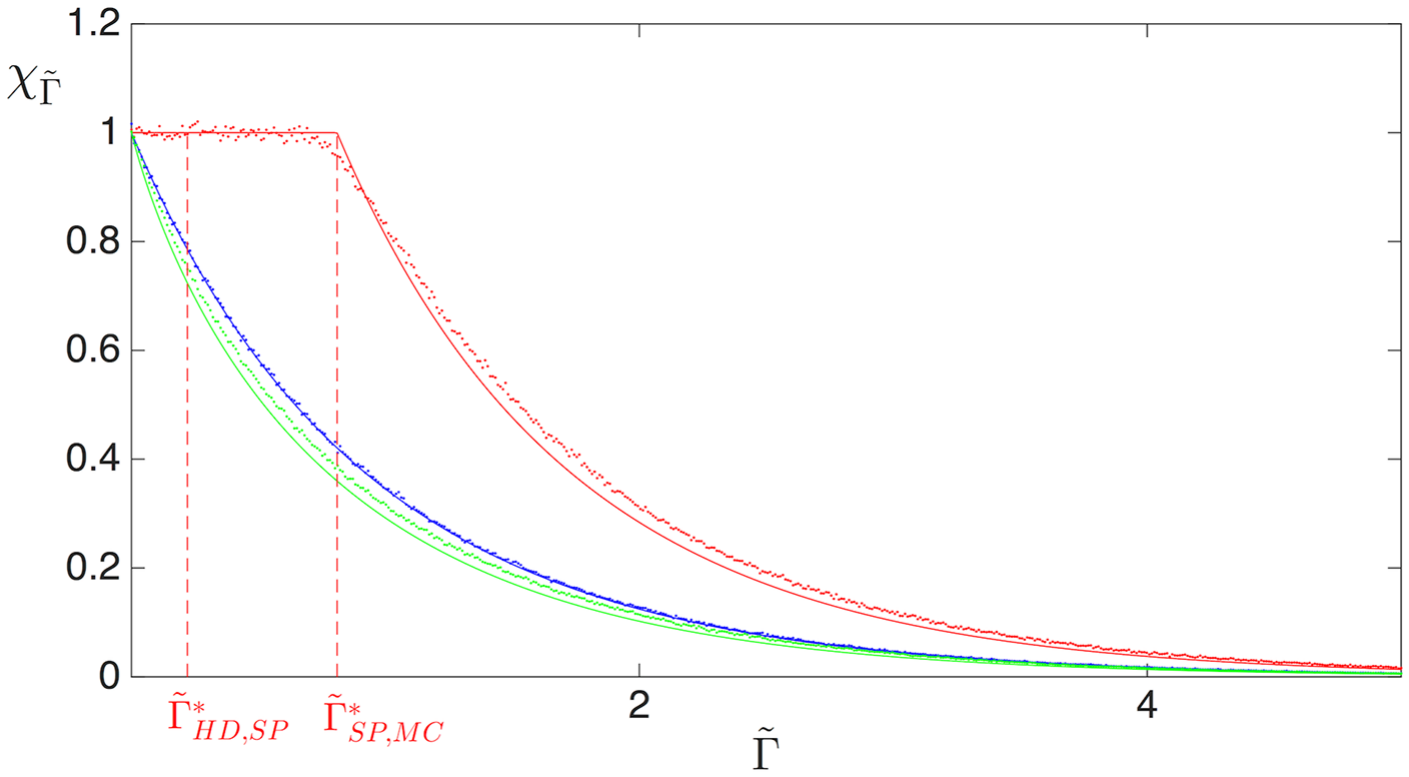
Resilience to drop-off for various choices of initiation and termination rates, computed analytically (solid lines) and numerically (dots) for *L* = 1. The blue line represents a mRNA sequence in the low density regime, with $\tilde{\alpha }=0.1$ $\tilde{\beta }=1$. The red line corresponds to a sequence starting in the high density regime with $\tilde{\alpha }=1$, $\tilde{\beta }=0.1$. The maximal current regime is represented by the green line with $\tilde{\alpha }=1$ and $\tilde{\beta }=1$.

Fig 11 shows $\chi_{\tilde{\Gamma }}$ depending on $\tilde{\Gamma }$ for mRNAs in three different phases: LD (blue), HD (red) and MC (green). The ribosome drop-off rate has a similar impact on both the LD and the MC regime, showing a striking decrease in the translation efficiency with the drop-off rate $\tilde{\Gamma }$. The HD phase, however, is crucially different: if an mRNA strand carries an HD phase, then the ribosome drop-off does not lead to a ribosome current loss at first. This also holds for the SP phase (for which the final section of the sequence is also in HD). This may be understood from the observation that in these phases the current is determined by the exit rate, implying that the density at the last site of the mRNA lattice is $\rho \left(L\right)=1-\tilde{\beta }$, irrespective of the presence or absence of ribosome drop-off in the bulk (see Eqs (22) and (23)). This reflects the fact that, since initiation is frequent, and ribosomes which drop off are effectively replaced, the impact of drop-off is compensated in this regime. Therefore, the protein synthesis rate of those mRNA strands whose dynamics is governed by ribosome queueing, i.e. those carrying HD and SP phases, is not affected by ribosome drop-off. The following section will show that this remains qualitatively correct even for more realistic models of the mRNA strand.

Fig 11 also shows, however, that this only holds up to a critical value of the drop-off rate, beyond which the drop-off resilience $\chi_{\tilde{\Gamma }}$ finally decreases. Indeed, the red curve corresponds to an mRNA which shows perfect resilience at first: its translation rate remains entirely unaffected by a small drop-off rate. Closer inspection shows that the mRNA first carries a HD phase (for very small $\tilde{\Gamma }$), but then undergoes a first transition from HD to SP (left vertical dashed line in Fig 11) as the drop-off rate $\tilde{\Gamma }$ is increased. This is followed by a second transition from SP to MC (right vertical dashed line in Fig 11), beyond which the resilience starts to decay. The sequence of transitions is easily visualised in Fig 10, where the red dashed line indicates the termination rate $\tilde{\beta }=0.1$ associated to this particular mRNA. Where this line crosses ${\tilde{\beta }}_{l}^{*}$ and ${\tilde{\beta }}_{u}^{*}$ (green and blue lines, respectively) indicates the values of $\tilde{\Gamma }$ at which the HD-SP and SP-MC transitions occur, corresponding to ${\tilde{\Gamma }}_{HD,SP}^{*}$ and ${\tilde{\Gamma }}_{SP,MC}^{*}$ in Fig 11, respectively.

To calculate the value ${\tilde{\Gamma }}_{HD,SP}^{*}$ we use the fact that the termination rate fulfills $\tilde{\beta }={\tilde{\beta }}_{l}^{*}$ for this particular value of $\tilde{\Gamma }$. Using Eq (29), this occurs at a ribosome drop-off rate

$${\tilde{\Gamma }}_{HD,SP}^{*}\left(\tilde{\beta }\right)=\frac{2\left(1-\tilde{\beta }\right)-1-\ln\left(2\left(1-\tilde{\beta }\right)\right)}{L}.$$ (32)

Analogously, we can find the critical value for the ribosome drop-off rate at which the mRNA subsequently switches from the SP to the MC phase, which occurs when $\tilde{\beta }={\tilde{\beta }}_{u}^{*}$. Using Eq 28 one finds

$${\tilde{\Gamma }}_{SP,MC}^{*}\left(\tilde{\beta }\right)=\frac{2\tilde{\beta }-1-\ln\left(2\tilde{\beta }\right)}{L}.$$ (33)

Hence, if $\tilde{\Gamma }$ exceeds ${\tilde{\Gamma }}_{SP,MC}^{*}\left(\tilde{\beta }\right)$ the mRNA can no longer sustain a HD phase, not even close to the exit site. This is the point beyond which the translation rate efficiency starts to decrease with the drop-off rate.

**Fig 12:**
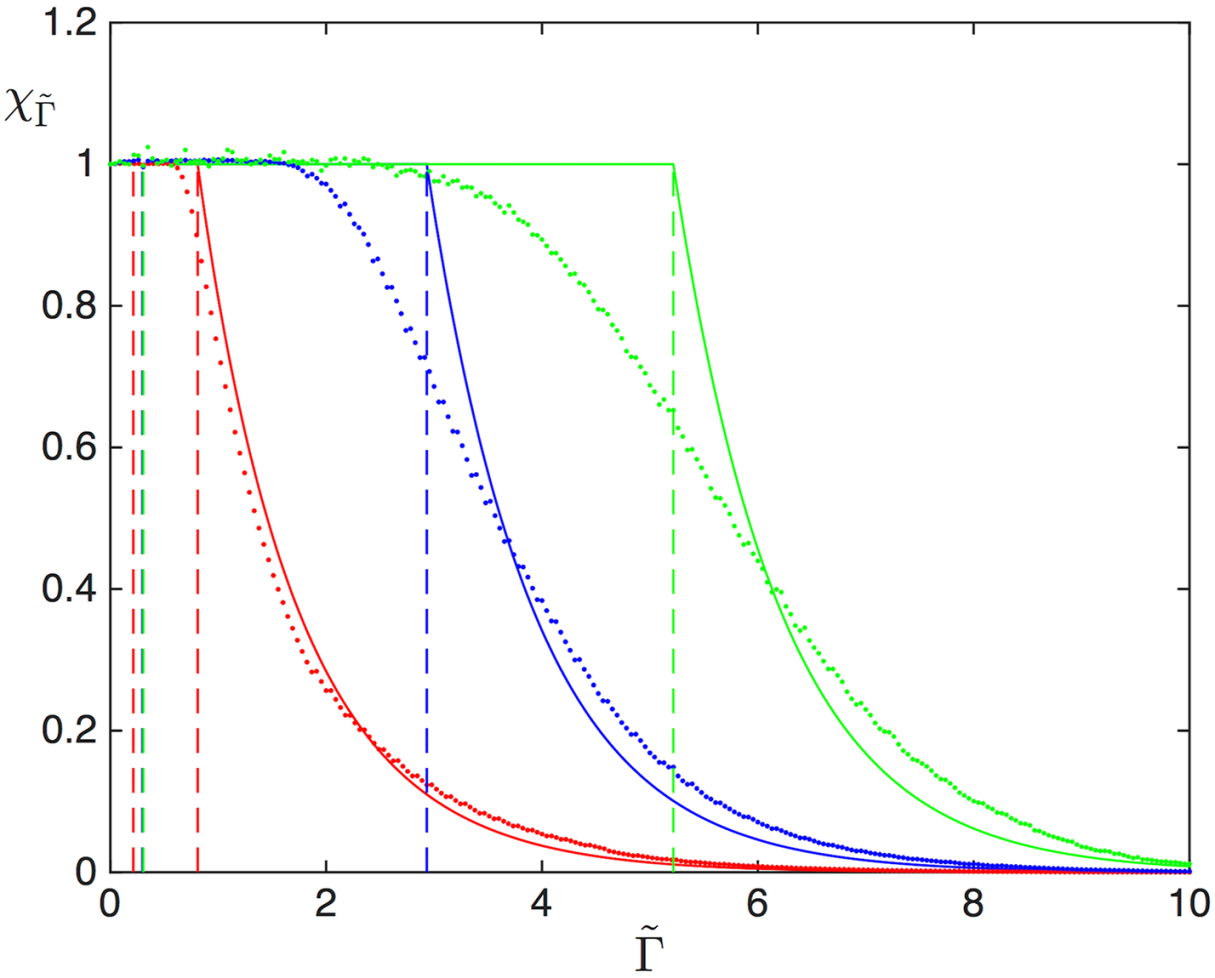
Resilience to drop-off, computed analytically (solid lines) and numerically (dots) for *L* = 1 and $\tilde{\alpha }=1$. Red: $\tilde{\beta }=0.1$, blue: $\tilde{\beta }=0.01$, and green: $\tilde{\beta }=0.001$. For these simulations, the transient time was 10^9^ iterations, followed by an integration time of 10^9^ iterations.

Importantly, it follows from this analysis that mRNAs with a slower termination rate $\tilde{\beta }$ are less susceptible to ribosome drop-off. This is illustrated in Fig 12, which represents the drop-off resilience $\chi_{\tilde{\Gamma }}$ for three mRNAs with different values of $\tilde{\beta }$, all of them starting in HD. We indicate with dashed lines the mean-field results and the mean-field critical values ${\tilde{\Gamma }}_{HD,SP}^{*}\left(\tilde{\beta }\right)$ and ${\tilde{\Gamma }}_{SP,MC}^{*}\left(\tilde{\beta }\right)$ for each of the mRNAs. Simulation results clearly confirm the fact that the translation rate remains unaffected by small drop-off rates, and mRNAs with large exit rates are first to suffer a drop in translation efficiency. As $\tilde{\beta }$ decreases, however, the disagreement between the mean-field expression and the simulations worsens. Increasing the transient and total integration time does not yield any improvement, and increasing the lattice size to *N* = 1000 leads only to a slight improvement. Simulations show that as $\tilde{\beta }$ decreases, correlations become important, and therefore the mean-field fails to correctly locate the value of $\tilde{\Gamma }$ at which the SP-MC transition occurs.

In conclusion, our model indicates that the translation rate of mRNAs carrying a HD phase are substantially more resilient to ribosome drop-off compared to other mRNAs carrying LD or MC phases.

### mRNAs with high elongation rates are more resilient to ribosome drop-off

**Fig 13:**
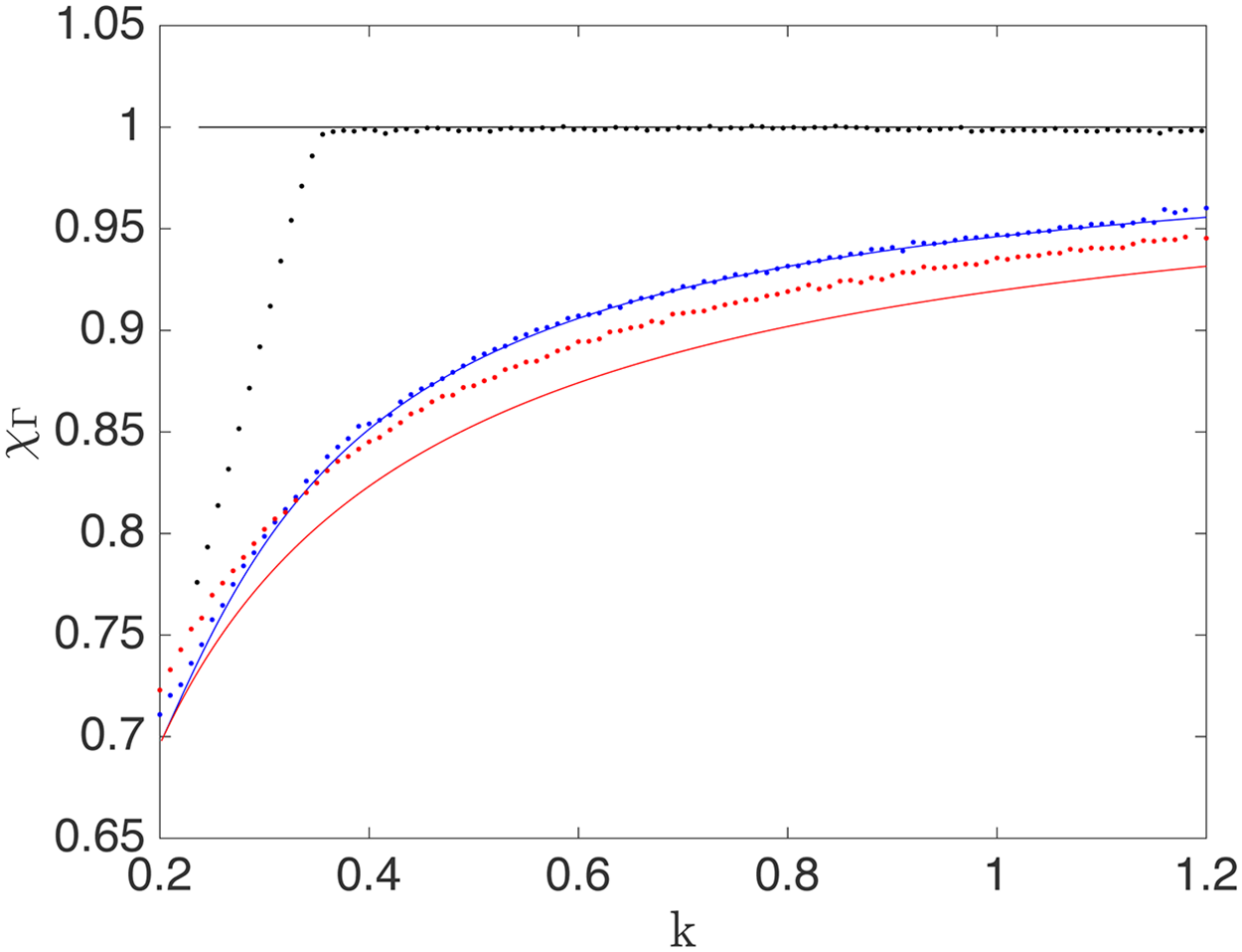
Drop-off resilience versus elongation rate analytically (solid lines) and numerically computed (dots) for *L* = 1 and Γ = 0.05. Blue (LD): *α* = 0.1, *β* = 1; Red (MC): *α* = 1, *β* = 1; Black (HD): *α* = 1, *β* = 0.1. For these simulations, the transient time was 10^7^ Gillespie iterations, followed by an integration time of 10^8^ iterations.

We next analyse how the resilience of mRNAs to ribosome drop-off is affected by the elongation rates *k*. We start by computing the resilience of a homogeneous mRNA sequence depending on its elongation rate *k*. We have seen in the previous section that mRNA sequences can be classified according to the phase they can carry, and the translation rate of mRNAs that sustain an HD phase is not affected at all by ribosome drop-off, i.e., they achieve the maximal value of resilience. We therefore analyse the effect of the elongation rate on the resilience for 3 different mRNA sequences, each one carrying an LD, MC or HD phase, respectively. The results are shown in Fig 13, where the solid lines show the analytical results and the dots represent the results from the numerical simulations. It is clearly seen that for both LD and MC, the resilience to drop-off increases monotonously with the elongation rate *k*. In contrast, for the sequence carrying HD, the resilience remains constant at the maximal possible value, *χ*_Γ_ = 1, as expected. Notice that the range of values of the elongation rate *k* for each curve is chosen so that the corresponding phase (LD, MC or HD) is sustainable with the given parameters. For example, for the chosen parameters shown in Fig 13, to be in LD we have to fulfil *α* < *k*/2; the condition for MC is *α* > *k*/2, and for HD we must ensure that *β* < *β*_*l*_, with *β*_*l*_ defined as in Eq (29) (where one must take into account that *β*_*l*_ depends on *k*).

The results obtained for the LD and MC phases are quite intuitive: high values of elongation rate lead to fast ribosome transit through the mRNA, and consequently, the probability that they are affected by drop-off is smaller. Hence, higher elongation rates lead to higher resilience. However, the results for the HD phase might appear counter-intuitive; since in HD ribosomes have longer dwell times due to queue formation, one might expect lower values for the resilience compared to the LD and MC cases. This can be understood, however, realising that in HD the translation rate is determined by the ribosome density at right boundary of the lattice. Due to the fact that the exit rate in this phase is more “limiting” than the drop-off rate, ribosome drop-off does not affect the translation rate value. Nevertheless, the probability for a ribosome to finish translation is indeed smaller in the HD phase than in the other phases (see ‘Supplementary Information’).

The numerical results for the MC phase show some discrepancy with respect to the analytical results. This is due to the higher correlations present in the MC phase with respect to the other phases, neglected in the mean-field approximation. Moreover, there is also a slight discrepancy between analytical and numerical results in the minimal value of the hopping rate *k* needed to sustain HD for the given *α*, *β*, and Γ parameters (*β* < *β*_*l*_, (Eq (29)). This indicates that there is a slight discrepancy between the mean-field prediction for the boundaries between HD and SP, as seen in Fig 8.

Taken together, these results suggest that mRNA sequences using fast codons are more resilient to ribosome drop-off than mRNA sequences containing slower codons. This is consistent with the long-standing observation that mRNAs whose usage of different synonymous codons is highly biased (using predominantly fast codons) code for proteins needed at a high level, such as ribosomal proteins and proteins involved in translation [47–49]. Hence, it suggests a regulatory mechanism by which such mRNAs not only have a higher translation rate, but they are also less vulnerable to ribosome drop-off.

### Application to real mRNA sequences

We now apply the ribosome drop-off model to mRNA sequences from *S. cerevisiae*. As mentioned previously, for such real mRNAs with a specific codon sequence, the elongation rate is in fact codon dependent. This amounts to inhomogeneous hopping rates along the lattice, according to the mRNA sequence. Here we analyse to which extent the conclusions drawn from the theoretical analysis in the previous sections, essentially based on mono-codon sequences, hold for real mRNA sequences.

To better match our model to the context of translation, we also account for the size of the ribosomes, by acknowledging that a ribosome should be taken to cover 9 codons [7]. In the language of our lattice model, this means that a particle (ribosome) now covers 9 lattice sites, although it still steps ahead by single sites. This question has been explored in several studies [50, 51]. Although it leads to a significant modification in the current-density relation, the qualitative phenomenology remains very similar. This refinement therefore serves to better represent the physiological conditions, but should not compromise the conclusions established above.

We have determined the hopping rates *k*_*i*_ associated to each of the codons in the sequence based on the corresponding tRNA abundance for *S. cerevisiae*, and used the estimated termination rate *β* = 18.03 *s*^−1^, as detailed in ‘Supplementary Information’. The value of the termination rate *β* is assumed to be the same for all mRNAs, since it depends on the concentration of available release factors. We now analyse the effect of two different values of the ribosome drop-off rate estimated in [29]: *γ* = 1.4 × 10^−3^ *s*^−1^, i.e. the value that has been estimated under physiological conditions, and *γ* = 5.6 × 10^−3^ *s*^−1^, estimated for ethanol intoxication conditions, both for *E. coli*. We then compare with results without drop-off (*γ* = 0) for reference. In [29] the values for the drop-off rate are given in units of ‘events/codon’. We transform them to units of ‘events/second’ by considering that the average speed of ribosomes is 10 codons/s. For example, 1.4 × 10^−4^ events/codon means that on average one ribosome needs to travel 1/1.4 × 10^−4^ codons before it drops off the mRNA. Equating this distance to the average ribosome speed times the average time between drop-off events allows calculating the latter, and therefore we obtain the frequency as the inverse of the average time between drop-off events. To our knowledge, there are no measurements of ribosome drop-off rate for yeast, and we thus adopt the same rates as for *E. coli*, since the mechanism of translation elongation is similar in prokaryotes and eukaryotes, and *E. coli* and yeast are both rapidly growing microorganisms found in environments where competition demands similar optimisation of the translation system [24, 25, 27, 28]. Moreover, we point out that we do not expect our results to change qualitatively even in the case that the drop-off rate *γ* turned out to be significantly different from the one estimated for *E. coli*, based on the results presented in Fig 11: as the resilience decreases monotonously with *γ*, all phases would be affected in a similar way. The only scenario for a qualitative change to occur would be for mRNAs containing an elongation bottleneck. For these, a substantially higher value of *γ* might preclude them from sustaining a HD phase, and it is only for mRNAs exhibiting a very severe elongation bottleneck that a HD regime might survive.

Our theoretical results in the previous section indicate that the impact of these ribosome drop-off rates strongly depends on the phase of the mRNA sequence under consideration: low density, high density or maximal current regime. But additionally, the effects of ribosome drop-off will clearly be stronger the longer the mRNA sequence is. The rescaled drop-off rate Γ = *Nγ*/*L* served to take this into account, precisely by making the results comparable whatever the length of the sequence. Here however, we focus on the effect of ribosome drop-off on translation in real mRNAs, and we thus reason in terms of the actual (non-rescaled) drop-off rate *γ*. We thus select three representative mRNA sequences from *S. cerevisiae*, all of them consisting of approximately 500 codons, and corresponding to the different regimes LD, HD and MC. For each sequence we choose the appropriate value of the initiation rate to ensure that it is in the corresponding phase (LD, HD and MC, respectively). These values do not correspond to the physiological initiation rate values; this simulation just illustrates the effects of drop-off depending on the phase for real mRNAs. In the next section we analyse the effect of drop-off for the *S. cerevisiae* genome under physiological values of the initiation rate.

**Fig 14:**
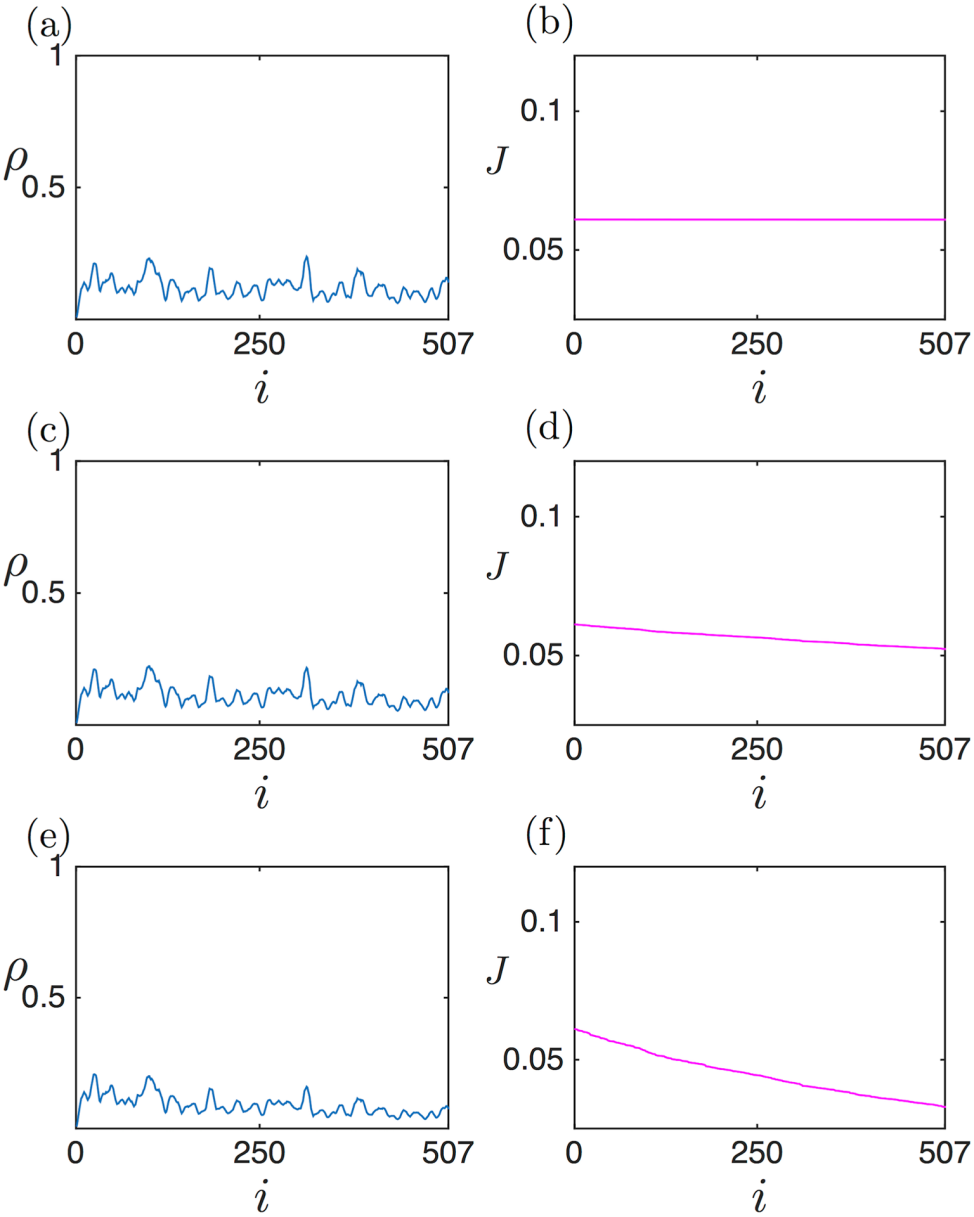
Density (a,c,e) and current (b,d,f) profiles for CDC7 (YDL017W), for *α* = 7 × 10^−2^ *s*^−1^, in a low density-like phase. In the density profiles we represent the coverage density of ribosomes, i.e., the probability for a codon to be covered by a ribosome. (a),(b): no ribosome drop-off *γ* = 0; (c), (d): physiological value of ribosome drop-off *γ* = 1.4 × 10^−3^ *s*^−1^; (e), (f): ribosome drop-off under ethanol stress *γ* = 5.6 × 10^−3^ *s*^−1^. The slope in the current profile clearly increases with the ribosome drop-off rate, and in the density profile the slight slope towards the 3’ is also reinforced by drop-off.

We first analyse the mRNA sequence of CDC7 (YDL017W), involved in origin firing and replication fork progression. At the initiation rate value of *α* = 7 × 10^−2^ *s*^−1^ this mRNA is expected to carry an LD phase. Fig 14 shows the results for the density and current profiles for this sequence, which clearly qualify as LD-like, subject to the expected disorder along the sequence. As the ribosome drop-off rate increases (middle and lower panels) we obtain density and current profiles which decrease along the lattice, as predicted by the theoretical results in the section ‘Density and Current Profiles’, and indeed the slope grows more important for higher *γ*. Moreover, the current at the first site of the mRNA remains constant as we increase *γ*, also in accordance with the theoretical results.

**Fig 15:**
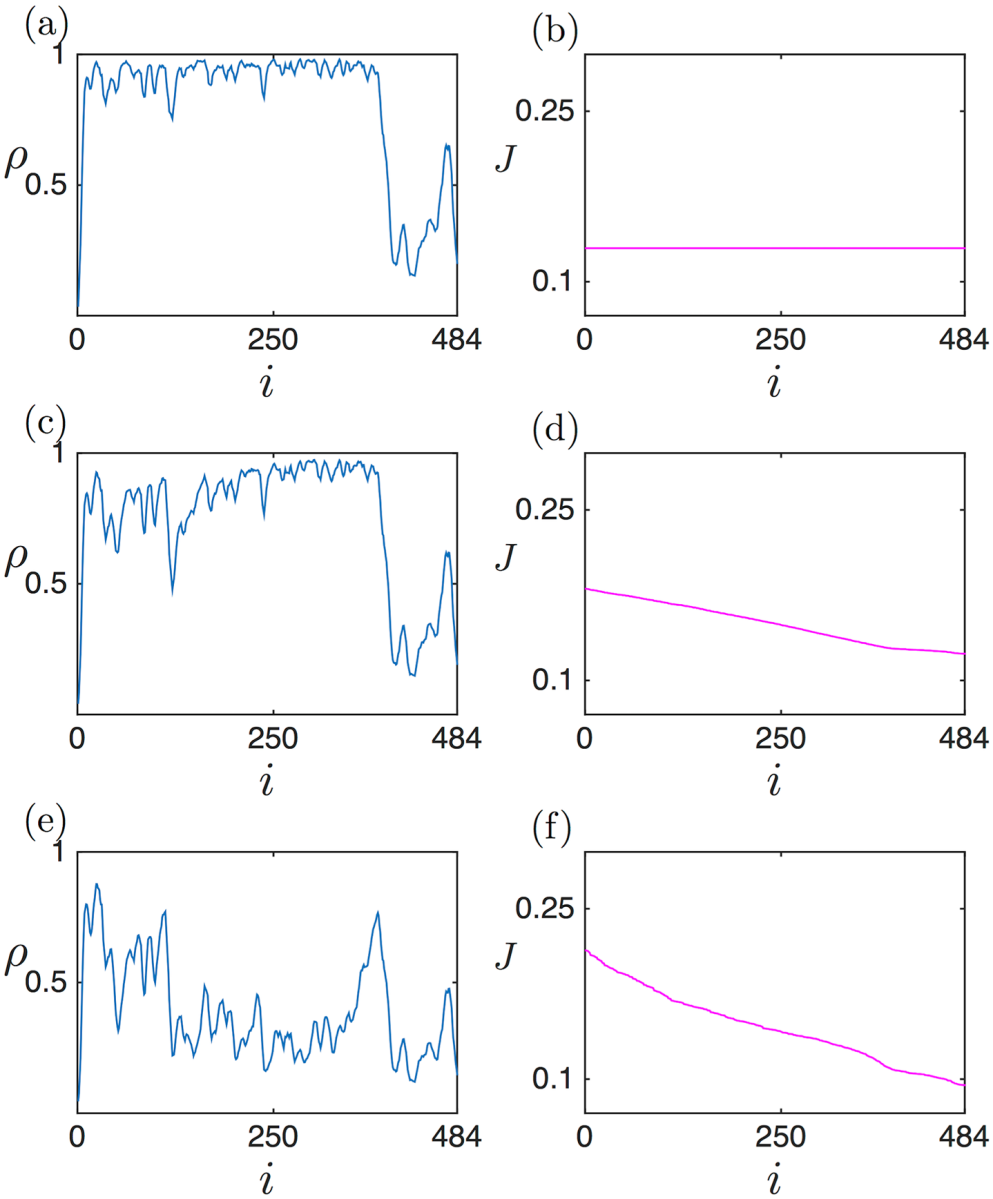
Density (a, c, e) and current (b, d, f) profiles for SLT2 (YHR030C), for *α* = 1*s*^−1^, a in high density-like phase. Details, including the values of *γ*, are as in Fig 14. (a),(b): no ribosome drop-off *γ* = 0; (c), (d): physiological value of ribosome drop-off *γ* = 1.4 × 10^−3^ *s*^−1^; (e), (f): ribosome drop-off under ethanol stress *γ* = 5.6 × 10^−3^ *s*^−1^. The increasing slope in the 5’ to the 3’ direction characteristic of the HD phase is clearly visible in panel (c). Moreover, panel (d) illustrates the marked increase in the current at the 5’ end, compared to the case without ribosome drop-off (see panel (b)), also characteristic of the HD phase.

As a second example we consider the mRNA sequence of SLT2 (YHR030C), a serine/threonine MAP kinase involved in regulating the maintenance of cell wall integrity, cell cycle progression, and nuclear mRNA retention in a heat shock. For a value of *α* = 1*s*^−1^ this sequence is in a high density-like regime. The corresponding density and current profiles are shown in Fig 15. The ribosome queue is clearly visible in the upper panels with no ribosome drop-off (*γ* = 0), where the density is almost 1 along the queue, before it drops dramatically after approximately codon 400, where the bottleneck in translation is caused by a cluster of slow codons. Then, for the physiological value of *γ* (Fig 15c), the density profile in the queue shows a slightly steeper slope from the 5’ to the 3’ end, reminiscent of the increasing density profiles obtained for HD sequences in the section ‘Density and Current Profiles’. The ribosome queue then starts to vanish for higher values of *γ*, corresponding to ethanol stress, as illustrated in Fig 15(e). Importantly, the current at the end of the lattice remains constant as we increase *γ* (compare Fig 15b and 15d), as predicted for sequences in HD, so that the current at the beginning of the mRNA increases with respect to the case without ribosome drop-off (compare panels b, d and f at the start codon, *i* = 0). This implies that the increased drop-off rate leads to a higher current at the beginning of the mRNA, again characteristic of mRNAs in HD, as shown in the section ‘Density and Current Profiles’. Furthermore, we see that for this sequence, the critical value of the drop-off rate beyond which the resilience starts decreasing is below the physiological *γ*, even though there are still clear signatures of an HD-like phase for the physiological *γ*. We expect this critical value of *γ* to be mRNA-dependent, determined by the severity of the elongation bottleneck present in the mRNA.

**Fig 16:**
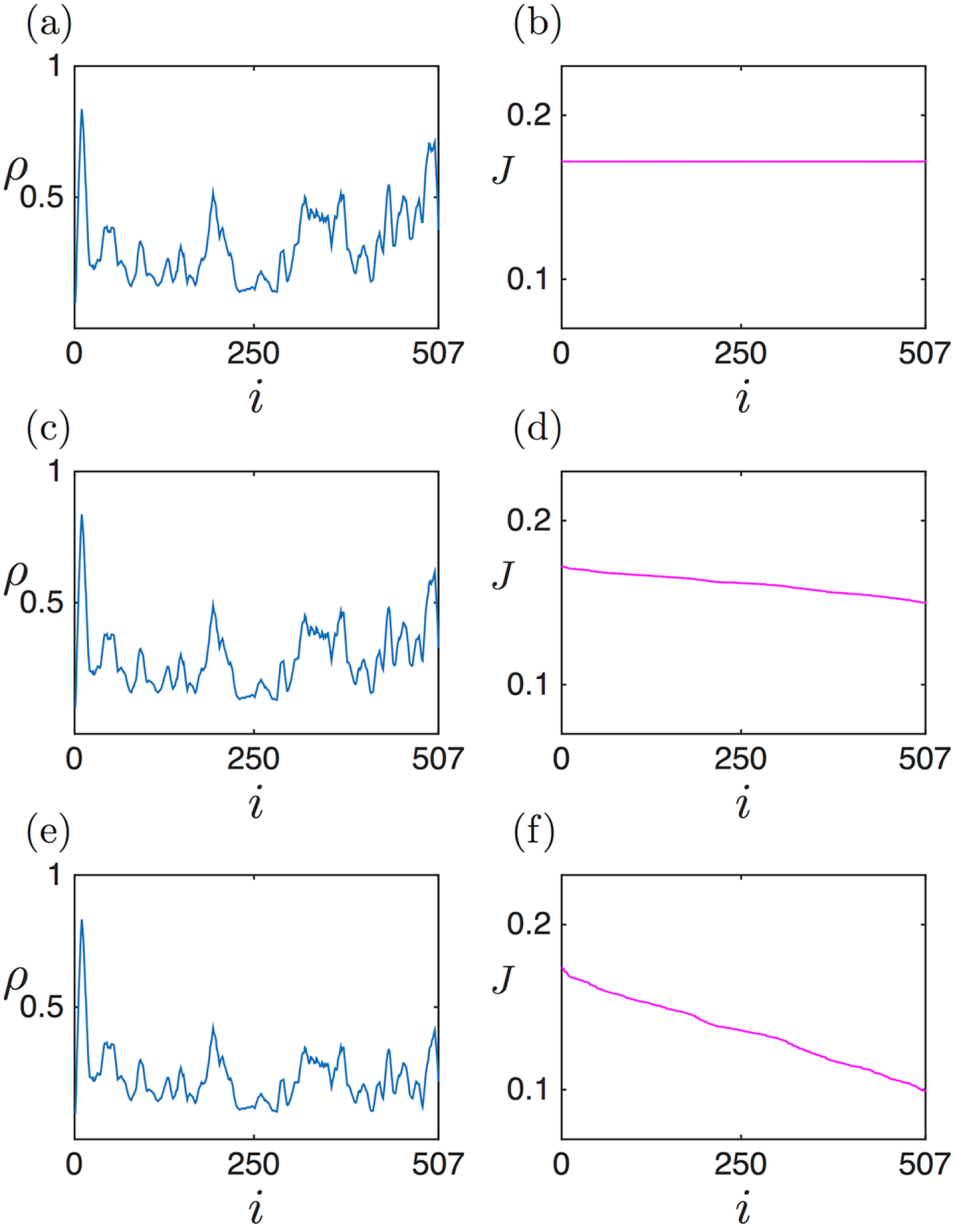
Density (a, c, e) and current (b, d, f) profiles for CRH1 (YGR189C), for *α* = 1*s*^−1^, in a maximal current-like phase. Details, including the values of *γ*, are as in Fig 14. (a),(b): no ribosome drop-off *γ* = 0; (c), (d): physiological value of ribosome drop-off *γ* = 1.4 × 10^−3^ *s*^−1^; (e), (f): ribosome drop-off under ethanol stress *γ* = 5.6 × 10^−3^ *s*^−1^. The current profiles clearly show an increase in the slope as ribosome drop-off increases, qualitatively similar to the effects predicted for sequences in MC.

Finally, we analyse the mRNA sequence of CRH1 (YGR189C), a chitin transglycosylase involved in chitin transfer in the cell wall. For a value of *α* = 1*s*^−1^ this sequence is in a MC-like regime. Fig 16 shows the effects of increasing ribosome drop-off on the density and current profiles. The current profiles (Fig 16b, 16d and 16f) clearly show a steeper slope as *γ* increases, qualitatively similar to the predictions for sequences in MC in the section ‘Density and Current Profiles’. The effects on the density profiles (Fig 16a, 16c and 16e) are not as obvious as on the current profiles, due to the inhomogeneous hopping rates. However, a decrease in the density can be observed when focussing on the 3’ end of the mRNA.

In order to quantify the effect of ribosome drop-off on each sequence, we calculate their individual drop-off resilience (defined in Eq 31), for each of the considered values of *γ*. The results are summarised in Table 1. As predicted by the theoretical results, we see that the mRNA in the HD-like phase, SLT2, is the least affected by ribosome drop-off: the loss is only 5% in translation rate under physiological conditions, compared to 14% and 13% for the other two mRNAs in LD and MC-like phases, respectively. The advantage of the HD-like phase with respect to ribosome drop-off is also clear under stress conditions, with a loss of only 27% in translation rate compared to 46% and 43% for the other two phases.

**Table 1:** Table summarising the results for the drop-off resilience $\chi_{\tilde{\Gamma }}$ (defined in Eq (31) for the 3 mRNA sequences CDC7 (LD), SLT2 (HD), and CRH1 (MC), and for three different values of *γ*.

mRNA	*γ* (*s*^−1^)		
	0 (no drop-off)	1.4 × 10^−3^ (physiological)	5.6 × 10^−3^ (ethanol stress)
CDC7 (LD)	1	0.8598	0.5391
SLT2 (HD)	1	0.9537	0.7316
CRH1 (MC)	1	0.8734	0.5787

Therefore, these results strongly suggest that the mechanism which has emerged from our theoretical analysis based on modelling single-codon mRNA is robust: also for real mRNA, the impact of drop-off rates is seen to be less important for HD-like sequences.

### Genome-wide analysis

Applying the ribosome drop-off model to 3 real mRNA sequences has illustrated the effect of drop-off, showing that its impact depends on the ribosome traffic regime (LD, HD and MC). We now evaluate the resilience to drop-off for the *S. cerevisiae* genome under physiological conditions and analyse how the biological function encoded by the mRNAs might be related to its resilience.

We start by estimating the physiological value of the initiation rate for each mRNA sequence by using genome-wide experimental data from [52] of ribosome density *ρ*_*ϕ*_ under physiological conditions (see ‘Supplementary Information’). For each mRNA sequence we then identify the physiological value *α*_*ϕ*_ of the initiation rate, using our ribosome drop-off model by requiring *ρ*(*α*_*ϕ*_) = *ρ*_*ϕ*_, i.e. we take the initiation rate to be the one which replicates the experimentally measured ribosome density (following the approach in [17]). The histogram of the *α*_*ϕ*_ values obtained in this way is shown in the ‘Supplementary Information’. With those values of *α*_*ϕ*_ it is possible to compare the model predictions for the translation rate to experimental data. To do this, we have calculated the Spearman’s rank correlation coefficient between $M\times J_{\alpha_{\phi }}$ and *P*, where *M* and *P* denote the experimentally measured mRNA and protein levels, respectively, taken from [53], and $J_{\alpha_{\phi }}$ denotes the translation rate estimated with our model. We obtain a value of 0.57, p-value< 10^−14^, yielding an improvement compared to other protein level predictors, such as the tAIc (Spearman’s rank = 0.38, p-value<10^−6^) [17, 53].

**Fig 17:**
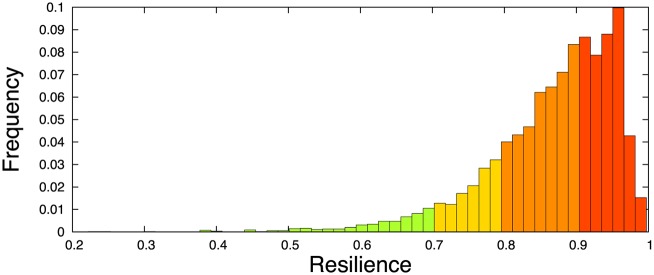
Histogram of ribosome drop-off resilience values *χ*_*γ*_ for the *S. cerevisiae* genome under physiological conditions. The colours of the different regions correspond to the following *χ*_*γ*_ intervals: green: *χ*_*γ*_ < 0.7; yellow: 0.7 ≤ *χ*_*γ*_ < 0.8; orange 0.8 ≤ *χ*_*γ*_ < 0.9, and red: *χ*_*γ*_ ≥ 0.9.

Using the ribosome drop-off rate *γ* = 1.4 × 10^−3^ *s*^−1^ as estimated under non-stress conditions [29], we can then exploit Eq (31) to compute the ribosome drop-off resilience for each mRNA sequence. The histogram of the resilience values *χ*_*γ*_ is represented in Fig 17. We see that the effect of the drop-off rate on the translation depends very much on the specific mRNA sequence, with values of the resilience ranging from 0.2 to approximately 1. Therefore, ribosome drop-off can lead up to an 80% decrease in the translation rate under physiological conditions, depending on the parameters characterising the mRNA sequence. Nevertheless, the mean resilience is 0.87 and the median resilience is 0.89, indicating that most mRNAs are only moderately affected by ribosome drop-off under physiological conditions.

We next investigate whether there is a correlation between the resilience to ribosome drop-off of an mRNA and the biological function of the protein it codes for. To address this question we divide the histogram into four regions, from small to high values of *χ*_*γ*_, and we run a GO enrichment analysis on each region (summarised in Table 2). This analysis was done using the software Gene Ontology Term Finder (http://go.princeton.edu/cgi-bin/GOTermFinder) developed at the Lewis-Singer Institute at Princeton. P-values are computed using the hypergeometric distribution, and the Bonferroni correction is applied to take into account multiple hypothesis tests [54]. We thus identify, for each of these regions, the GO categories which are significantly over-represented. The following observations emerge:

**Table 2:** Table summarising the results for Gene Ontology (GO) enrichment analysis for the 4 different regions of the histogram of the ribosome drop-off resilience values (Fig 17) obtained for the *S. cerevisiae* genome.

Region	*χ*_*γ*_ range	GO term	P-value
green	[0, 0.7)	biological regulation	1.47e-26
		response to stimulus	9.95e-25
		cell communication	1.15e-11
		chromosome organisation	1.16e-10
		regulation of intracellular signal transduction	1.73e-10
yellow	[0.7, 0.8)	biological regulation	6.14e-40
		regulation metabolic process	1.93e-20
		regulation of nitrogen compound metabolic process	5.55e-17
		response to stimulus	6.40e-17
		cell cycle process	2.76e-15
orange	[0.8, 0.9)	organic substance metabolic process	1.99e-23
		primary metabolic process	3.91e-20
		catabolic process	4.24e-09
		oxoacid metabolic process	7.79e-09
		cellular amino acid metabolic process	6.47e-07
		lipid metabolic process	1.73e-07
red	(0.9, 1]	cytoplasmic translation	3.08e-22
		ribosome assembly	2.14e-05
		ribosomal small subunit assembly	0.00101
		rRNA export from nucleus	0.00360
		rRNA transport	0.00360

In the two regions which correspond to the mRNAs with the lowest ribosome drop-off resilience (coloured green and yellow, respectively), the dominant GO categories are related to biological regulation, response to stimulus, regulation of metabolic process, signal transduction and cell cycle. In the next region (orange), with a fairly high value of drop-off resilience, the salient GO categories are related to central metabolism processes. Finally, in the region with the strongest resilience against drop-off (red), a highly disproportionate number of mRNAs code for proteins involved in cytoplasmic translation, ribosome assembly, as well as rRNA export and transport. Hence, in essence, mRNA sequences coding for translation and metabolism-related proteins are predominant amongst those sequences with high resilience, whereas mRNAs with low drop-off resilience mainly code for regulatory proteins and those involved in stress responses. A GO enrichment analysis taking the same number of genes from each of the four highlighted histogram regions yields results in agreement with the results obtained with the whole sections (see Supplementary Tables).

It is interesting to link this observation to the fact that mRNA sequences relevant to translation and central metabolism are known to be abundant under physiological conditions, whereas those concerning stress-regulation are present at much lower levels only. This resullt is consistent with the finding in [22] that the ribosome drop-off effect is weaker for mRNAs coding for highly expressed genes, even though their analysis was based on only 10 genes. There, the authors argue that highly expressed genes consume more ribosomes, due to their higher mRNA levels, and that it is therefore beneficial for the cell to optimise their codon sequence in order to minimise losses in translation due to ribosome drop-off. This analysis therefore raises the question whether resilience is an important feature of highly expressed genes, which would point to an evolutionary selection mechanism operating to optimise their resilience to ribosome drop-off.

Identifying the biological functions which the classes of mRNA predominantly refer to also provides an indication of the origin of the high resilience of translation-related mRNA sequences. Indeed, these mRNAs have been shown to be highly codon-biased, i.e. they have been optimised to ensure high elongation rates [47–49]. From our mathematical analysis in the previous section, we know that high elongation rates help to minimise the effect of drop-off (see Fig 13). The argument thus points to the bias towards fast codons as the origin for high resilience.

All in all, the analysis on a genome-wide scale reveals several properties which appear to be linked to resilience and attributes a coherent set of properties to mRNAs with specific gene ontologies. One one hand, as already stated, the value of the resilience *χ*_*γ*_ correlates with the elongation rate *k*, in agreement with our observation above that high elongation rates help to reduce the impact of drop-off. On the other hand, the resilience *χ*_*γ*_ also correlates with the physiological value of the initiation rate *α*_*ϕ*_ (Pearson correlation coefficient *R* = 0.42, p-value *p* = 3.36 × 10^−232^), and the physiological value of the translation rate (*R* = 0.58, p-value *p* < 2.2251 × 10^−308^). Interestingly, for sequences in LD phase, the resilience to drop-off slightly decreases as *α* increases (see Supplementary Information), but the positive effect of high elongation rates on the resilience *χ*_*γ*_ is stronger. Hence, even though mRNAs using fast codons tend to have high initiation rates, they exhibit a higher resilience to ribosome drop-off compared to mRNAs containing slow codons.

All these observations support the fact that proteins involved in translation and central metabolism are known to be highly expressed under physiological conditions, in contrast to regulatory and stress-related proteins. Consequently, mRNAs coding for highly abundant proteins are typically characterised by high elongation and initiation rates, leading to a high translation rate. This is indeed confirmed on the genome-wide analysis of physiological *α*-rates, accompanied by a GO enrichment analysis (shown in the ‘Supplementary Information’).

From an additional angle it is also interesting to investigate how the length of the mRNA sequences is linked to their resilience. The resilience values *χ*_*γ*_ are highly anti-correlated with the mRNA length (*R* = −0.97 and p-value *P* < 2.2251 × 10^−308^). In view of the drop-off model, this behaviour can again be rationalised: the ribosome drop-off mechanisms, and therefore the drop-off rate *γ*, are essentially the same for all mRNAs. Therefore the impact of drop-off will be larger for longer mRNAs (see more detailed discussion in ‘Supplementary Information’). Accounting for ribosome drop-off might therefore offer an explanation for the long-standing observation that the average ribosome density on mRNAs in anti-correlated with the mRNA length, as experimentally measured in [7, 55], and still under debate [11, 17, 29].

## Discussion

Recent analysis of ribosome profiling data has shown that ribosome drop-off is statistically significant under physiological conditions, and that it becomes particularly important when cells are stressed, such as under amino acid starvation or ethanol intoxication [29]. These results highlight the need for a theoretical framework to analyse the effects of ribosome drop-off both on translation efficiency and on ribosome density profiles. Here we have studied a mathematical model to quantify and predict the effects of ribosome drop-off on translation. Our model is based on the extensively used Totally Asymmetric Simple Exclusion Process (TASEP) model for translation [17, 36, 37, 46, 50], but it incorporates the fact that ribosomes can drop off the mRNA at any codon with a rate *γ*. It can be considered as an adaptation of the TASEP with Langmuir kinetics [39], for which particles can both bind and unbind anywhere in the lattice, in the limit of a vanishing binding rate. By comparing our model with the conventional TASEP model we were able to analyse and quantify the effects of ribosome drop-off, thereby providing a tool to analyse and interpret ribosome profiling data.

Treating the mRNA lattice in a continuous approximation has lead to analytical mean-field expressions for the ribosome density and current profiles in all possible dynamical regimes: low density (LD), high density (HD), maximal current (MC) and shock phase (SP), showing good agreement with stochastic simulations. In LD and MC the ribosome density decreases along the mRNA sequence, from the 5’ to the 3’ end, but the density profile *increases* in the HD phase. The SP is characterised by a coexistence of lattice domains where the ribosome density is low (LD) or high (HD or MC). In contrast to the conventional translation model without ribosome drop-off, the position of the domain wall separating those coexisting regimes is fixed, analogously to the TASEP with Langmuir kinetics [39]. The resulting analytical expressions for the phase boundaries separating the different phases show good agreement with simulations. One direct implication of these results is that the HD phase cannot be sustained anymore once the ribosome drop-off rate reaches a critical value, which we have determined analytically. Beyond this critical drop-off rate the SP phase becomes less prominent in the phase diagram, which is now dominated by LD and MC phases.

The determination of the phase boundaries is of biological relevance, since the traffic dynamics on a specific mRNA is determined by the various rates applicable to initiation, elongation and termination. Those rates are strongly dependent on the experimental conditions (e.g. the initiation rate is directly related to availability of ribosomes, which are downregulated when cells are starved for nutrients). Usually, a change in those experimental conditions will only have a small effect, but whenever it leads to the ‘crossing’ of a phase boundary, it can have dramatic consequences for the traffic dynamics on a specific mRNA. Here, we have shown how the values of the respective rates at which one crosses these phase boundaries depend on the value of the drop-off rate. Predictions for the rates at which we cross a phase boundary can be validated by experiments, e.g. by changing elongation and initiation rates (e.g. the sup70-65 and gcn2 kinase mutants, respectively, used in [56]). Concerning the possibility of an increasing density profile, we do not believe this to be very common, in particular since the initiation rate *α*_*ϕ*_ is typically small compared to the elongation rates under physiological conditions. However, our recent experimental results indicate that ribosome queues can be induced as a result of elongation bottlenecks, due e.g. to depletion of charged levels of tRNAs [56]. Therefore, increasing ribosome density profiles, if detected, could be explained as resulting from the presence of ribosome queues together with ribosome drop-off.

We have then introduced the *resilience* to drop-off as the ratio between the translation rate in the presence and in the absence of drop-off, respectively, and which characterises the extent to which translation can be maintained despite ribosome drop-off. We have found that this resilience strongly depends on the mRNA sequence. Importantly, mRNAs carrying a HD phase (mRNAs containing a bottleneck in the middle or at the end of the lattice) are substantially less affected by ribosome drop-off than mRNAs carrying an LD or MC phase. Moreover, the more severe the bottleneck in those mRNAs (caused e.g. by slow codons towards the 3’ end), the more resilient the mRNA to ribosome drop-off will be.

We have then illustrated the effect of ribosome drop-off by applying a refined version of the ribosome drop-off model, accounting for codon-dependent elongation rates as well as the extended footprint of ribosomes. We have numerically studied 3 representative mRNA sequences from *S. cerevisiae*, choosing an initiation rate to establish LD-, HD- and MC-like phases respectively [17]. We have calculated the corresponding ribosome density profiles without ribosome drop-off (*γ* = 0), as well as the physiological value of *γ* and the value corresponding to acute ethanol stress. The results show that the ribosome density profiles qualitatively resemble those obtained for simplified mono-codon mRNA sequences used for our analytical model: this validates the conclusions drawn from the theoretical analysis, which thus remains applicable to real mRNA sequences.

Specifically, the results confirm that the translation rate of the mRNA sequence in a HD-like phase is substantially more resilient to ribosome drop-off in stress situations when compared to the mRNA sequences in LD and MC-like phases (27% drop of translation rate for the HD-like mRNA, compared to 43% and 46% drop of the other two). To underline the interest of this observation we recall that the type of phase transition that can take place on a given mRNA (LD-HD or LD-MC) significantly correlates with the gene ontology classification of the encoded protein [17]. In general terms, an mRNA sequence carrying slow codons towards the 3’ end undergoes an LD-HD-like transition as the initiation rate is increased. In contrast, an mRNA without slow codons, or with slow codons predominantly at the 5’ end, leads to an LD-MC-like transition. Intriguingly, the drop-off resilience could thus constitute an advantageous mechanism for the cell under stress, when regulatory proteins need to be produced. To understand this mechanism, two further observations are needed. First, the ribosome drop-off rates are known to be increased under stress [29]. Second, it has also been found that those mRNAs which can reach the HD phase predominantly code for regulatory proteins [17]. In the light of our findings, one would thus expect stress would push those mRNA strands which code for regulatory proteins into higher resilience to ribosome drop-off. Importantly, the resilience of mRNAs in HD remains unchanged upon further variations in the drop-off rate (see the plateau in Fig 11). This might be advantageous under stress situations, since the production of stress-related proteins would be maintained at maximal possible translation rate, ensuring robustness with respect to fluctuations in the environment.

Finally, we have performed a genome-wide analysis of the effects of ribosome drop-off in *S. cerevisiae*. We have first estimated the physiological value of the initiation rate for each mRNA, using literature data for the ribosome density on each mRNA in conjunction with the ribosome drop-off model. Based on these values of the initiation rates we have then calculated the ribosome drop-off resilience for each mRNA sequence under physiological conditions, and run a Gene Ontology (GO) analysis to correlate the resulting drop-off resilience with the biological function encoded by the mRNA. In general terms, mRNAs coding for proteins involved in translation, ribosome biogenesis and central metabolism present a high resilience to ribosome drop-off, in contrast to regulatory proteins involved in stress responses. This result therefore suggests that highly expressed genes might have evolved to be resilient to ribosome drop-off when conditions are favourable. As argued by the authors in [22], this might constitute an evolutionary mechanism by which the cell minimises losses in translation for mRNAs coding for highly expressed genes, since their translation utilises large numbers of ribosomes.

A next step in understanding the effect of ribosome drop-off on translation would be to estimate the values for the initiation rate for each mRNA under stress conditions. This estimation would have to be based on corresponding genome-wide measurements of ribosome densities, and would allow us to see whether mRNAs encoding regulatory proteins indeed increase their initiation rate upon stress. There are experimental observations indicating that translational resources, and in particular ribosomes, are redistributed among different mRNAs under stress conditions [6]. Redistribution causing an increase in initiation rate and therefore a transition to an HD-like phase would engender resilience to ribosomal drop-off, acting as an adjunct to initiation-based mechanisms of translational control of gene expression.

One way of addressing these questions is through the notion of limited resources, acknowledging that a finite pool of ribosomes in a cell is shared by all RNAs. The corresponding model thus aims at characterising individual initiation rates are affected by the competition for recruiting ribosomes [57]. Incorporating this competition, which couples the translation of different mRNAs, in the drop-off model might reveal yet more subtle mechanisms for regulation.

In conclusion, we have introduced a mathematical model to describe the effects of ribosome drop-off, which provides the theoretical framework to analyse ribosome profile data. This analysis has revealed that the effects of ribosome drop-off are mRNA-specific, with both composition and configuration of codons strongly affecting the loss of translation due to ribosome drop-off. Moreover, our results suggest that the resilience to ribosome drop-off of mRNAs might change upon stress due to redistribution of translational resources affecting their initiation rates. Therefore, resilience to ribosome drop-off might play an important role in gene regulation. A next step will be to analyse ribosome profiling data at the single gene level and compare the ribosome density profiles with the model predictions. Moreover, our model provides a tool to address a series of open problems in translation, such as the anti-correlation between ribosome density and length of the mRNA [7, 11, 17, 29, 55], and the effects of high level recombinant protein production and the corresponding consequences in terms of ribosome drop-off.

## Supporting information

S1 Text Supplementary information. This file contains 5 subsections, providing additional details on the (1) Discretisation of the mRNA Lattice; (2) Ribosome Drop-Off Effects Depending on mRNA Length; (3) Ribosome Drop-Off Effects Depending on Initiation Rate; (4) Estimation of Elongation Rates; (5) Genome-Wide Estimation of Physiological Values of Initiation Rates.
(PDF)

S1 Table Gene Ontology enrichment analysis according to initiation rate value. This table contains the results from the GO enrichment analysis performed for the different regions of the histogram of the physiological value of the initiation rates for *S. cerevisiae*. Different sheets correspond to different regions of the histogram and different GO analysis: process, function and component.
(XLSM)

S2 Table Gene Ontology enrichment analysis according to resilience value. This table contains the results from the GO enrichment analysis performed for the different regions of the histogram of the resilience to ribosome drop-off for *S. cerevisiae*. Different sheets correspond to different regions of the histogram and different GO analysis: process, function and component.
(XLSX)

S3 Table Elongation rates. List of tRNAs, with corresponding anticodons and codons, Gene Copy Number of the tRNAs (GCN) and estimated elongation rate (1/*s*) for each codon, as explained in S1 Text.
(XLSX)

S4 Table Complete list of genes used in the genome-wide analysis. Systematic gene name, reference number, number of codons, measured value of ribosome density (experimental data), estimated physiological value of initiation rate and calculated value of ribosome drop-off resilience.
(XLSX)

S5 Table Gene Ontology enrichment analysis according to resilience value, using same number of genes in each section of the histogram. Different sheets correspond to different regions of the histogram.
(XLSM)
