# Supplementary material for: Novel mRNA-specific effects of ribosome drop-off on translation rate and polysome profile
Source: PLoS Comput Biol. 2017 May 30;13(5):e1005555. doi: 10.1371/journal.pcbi.1005555 (PMC5469512; doi:10.1371/journal.pcbi.1005555)
Supplement: S1 Text — This file contains 5 subsections, providing additional details on the (1) Discretisation of the mRNA Lattice; (2) Ribosome Drop-Off Effects Depending on mRNA Length; (3) Ribosome Drop-Off Effects Depending on Initiation Rate; (4) Estimation of Elongation Rates; (5) Genome-Wide Estimation of Physiological Values of Initiation Rates. (PDF) [file pcbi.1005555.s001.pdf]

SUPPLEMENTARY INFORMATION: MRNA-SPECIFIC EFFECTS OF RIBOSOME DROP-OFF

P BONNIN, N KERN, N T YOUNG, I STANSFIELD, MC ROMANO

Contents

1. Discretisation of the mRNA Lattice 1
2. Ribosome Drop-Off Effects Depending on mRNA Length 3
3. Ribosome Drop-Off Effects Depending on Initiation Rate 5
4. Estimation of Elongation Rates 5
5. Genome-Wide Estimation of Physiological Values of Initiation Rates 8
References 9

1. Discretisation of the mRNA Lattice

We now address how the choice of discretisation for the lattice affects the simulation results, and how they compare with the analytical results derived in the main body of the manuscript. We therefore calculate the translation rate (current at the right boundary $J(L)$) of an mRNA with homogeneous elongation rate and ribosome footprint 1 for each of the LD, HD and MC phases, depending on the discretisation $\varepsilon = L/N$ used. In order to be able to compare lattices with different discretisations, we must keep the rescaled drop-off rate $\Gamma = \gamma/\varepsilon$ constant, as explained in the section 'Density and Current Profiles' of the main manuscript. Therefore, the analytical result for $J(L)$ will be independent of ε, since the expressions for the density and current profiles depend on Γ only, rather than on γ and ε separately. However, the results of simulations corresponding to the discrete system can depend on the chosen ε.

For this purpose, we fix L and increase the value of N within the interval $[10, 5000]$, which contains the interval of number of codons of the *S. cerevisiae* transcriptome (the shortest mRNA has 25 codons, and the longest one has 4991 codons). Then, for each value of N we calculate $J_{LD}(N)$, $J_{HD}(N)$ and $J_{MC}(N)$, as shown in Fig 1.

The solid lines indicate the analytical results, and the dots show the results from numerical simulations (LD (blue), HD (red) and MC (black)). As expected,

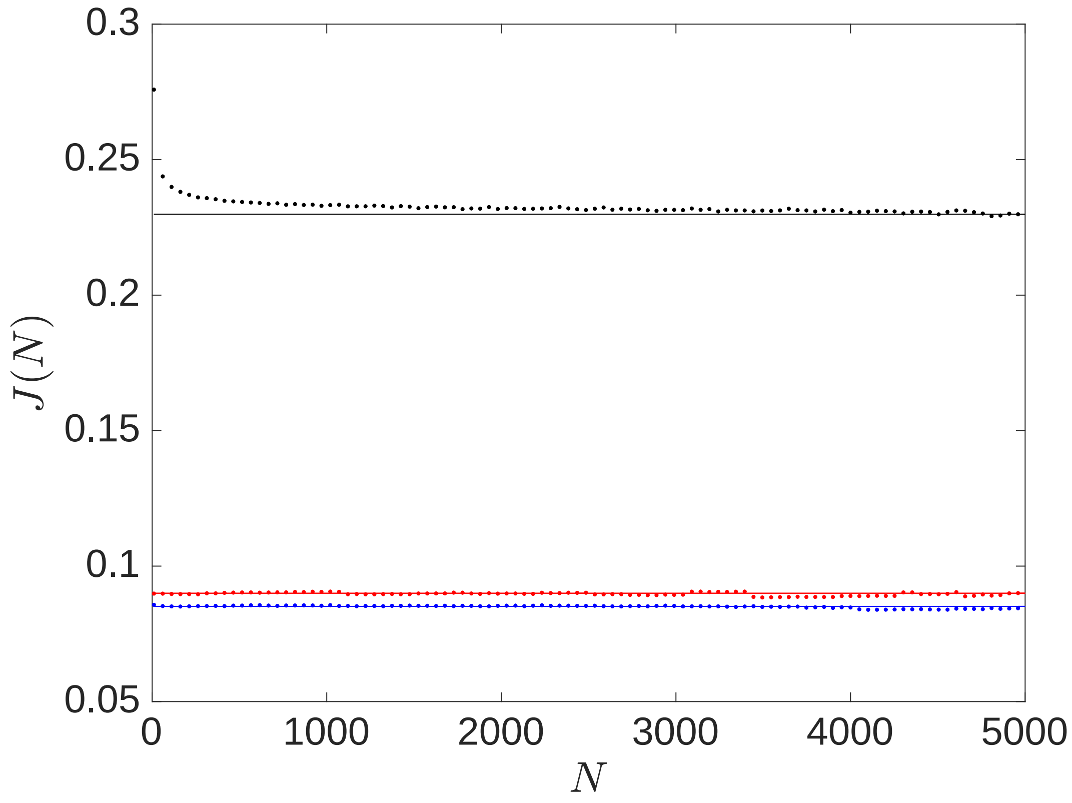

FIGURE 1. Translation rate $J(N)$ as a function of the discretisation used for the mRNA sequence with number of codons N and fixed length L, computed analytically (solid lines) and numerically (dots) for $L = 1$, $\Gamma = 0.05$, $\gamma = \varepsilon\Gamma$ and $k = 1$. Blue (LD): $\alpha = 0.1$, $\beta = 1$; Red (HD): $\alpha = 1$, $\beta = 0.1$; Black (MC): $\alpha = 1$, $\beta = 1$. For these simulations, the transient time was 10^7 iterations, followed by an integration time of 10^8 iterations.

the values of the analytical expressions for $J(L)$ are independent of ε, or alternatively, N. The values of the numerical results coincide very well with the analytical expressions for the LD and HD phases for all values of N. However, the values of $J_{MC}(N)$ deviate slightly from the analytical result for low values of N (coarse-grained discretisation), and it is seen to converge to the analytical value as N increases, i.e, for refined discretisations. This result therefore validates the continuous approximation adopted in the paper, showing that even for quite a coarse-grained discretisation of the mRNA lattice, the results obtained for the discrete system agree well with the results derived for the continuous system.

2. Ribosome Drop-Off Effects Depending on mRNA Length

One clearly expects the translation of longer mRNAs to be more strongly affected by ribosome drop-off than the one of shorter sequences. To analyse how the impact of ribosome drop-off scales with mRNA length, we compute the probability $P(L)$ for a ribosome to finish translation as a function of the sequence length L. This probability can be computed by calculating the ratio of the current of ribosomes at position L of the lattice, normalised by the current of ribosomes at position 0, i.e.,

$$P(L) = \frac{J(L)}{J(0)}. \tag{1}$$

It can be determined using

$$J(x) = k \lim_{\varepsilon \to 0} [\rho(x)\ (1 - (\rho(x) + \varepsilon \rho'(x)))] = k\, \rho(x)(1 - \rho(x)), \tag{2}$$

within the framework of the continuous approximation. We thus expect this probability $P(L)$ to depend on the phase sustained by the mRNA, given that the expressions for $J(x)$ differ between these phases. Indeed, by using the expressions for the ribosome density depending on x for each of the phases, $\rho_{LD}(x)$, $\rho_{HD}(x)$ and $\rho_{MC}(x)$ from the main manuscript and substituting them into Eq (2), we compute the probability $P(L)$ for an initiating ribosome to finish translation in the different phases (Fig 2). The solid lines indicate the analytical results, and the dots show the results from numerical simulations (LD (blue), HD (red), MC (black)). The correspondence between the continuous and discrete system is made by keeping both Γ and γ constant and increasing N proportionally to L, according to $N = \frac{\Gamma}{\gamma} L$.

As expected the probability to finish translation decays monotonously with the length L of the sequences whatever the transport phase. Interestingly, the HD case shows the strongest decay. This is due to the higher dwell time of ribosomes on the mRNA caused by the queue: ribosomes are more likely to undergo drop-off, and a smaller fraction of the initiated ribosomes make it to the stop codon, compared to the LD and MC cases. Notice that the analytical curve for $P(L)$ for the HD case finishes at a critical value L_c of approximately $L_c = 1.3$ for the parameters chosen. Beyond this length L_c the HD phase can no longer be sustained on the mRNA at the given values of Γ, k, α and β. This is in good agreement with the numerical results, which show that there is a plateau in $P(L)$ versus L, which starts at L_c. Ultimately the curve then merges with the one corresponding to the MC phase. This indicates that, as we keep increasing L, the lattice undergoes a (discontinuous) transition HD to MC, which passes through a coexistence region (the shock phase), the latter corresponding to the plateau. The effect of increasing L is thus equivalent to the effect of increasing the drop-off rate Γ, and therefore, it can cause phase transitions. Also notice that the plateau in $P(L)$ during the SP is directly due to the fact that $\rho(0) = \alpha/k$ and $\rho(L) = 1 - \beta/k$ in SP, independently

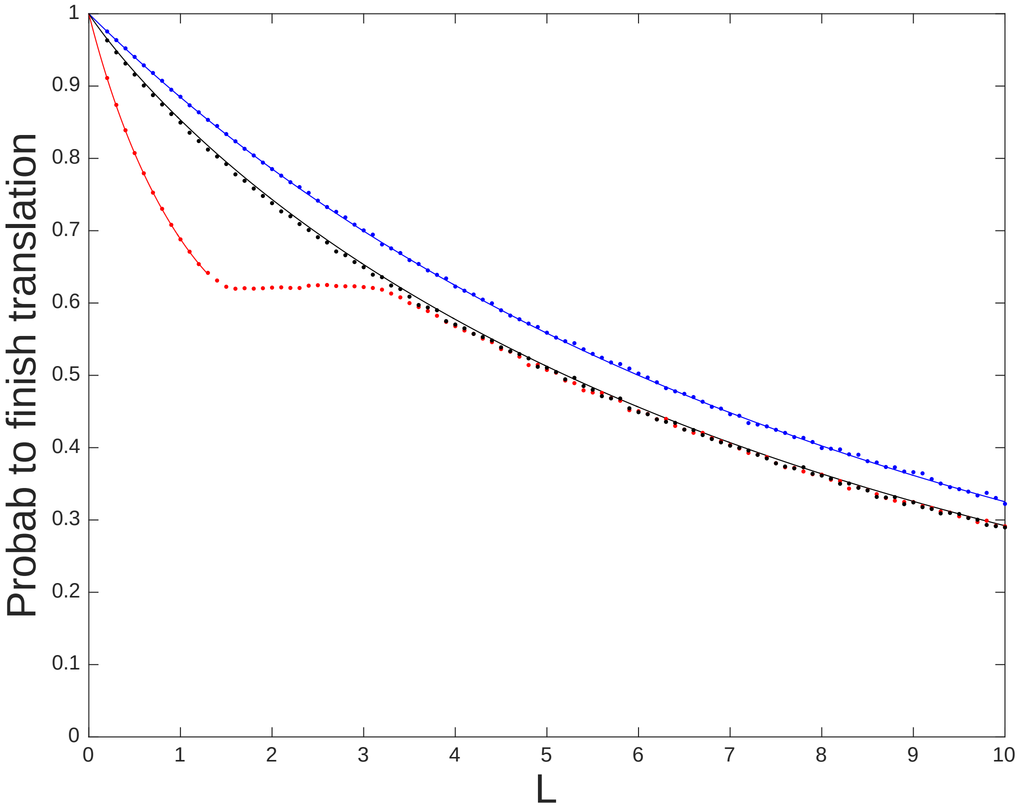

Figure 2. Probability $P(L)$ for an initiating ribosome to finish translation as a function of the mRNA sequence length L, computed analytically (solid lines) and numerically (dots) for $\Gamma = 0.1$, $\gamma = 2 \times 10^{-4}$ and $k = 1$. Blue (LD): $\alpha = 0.2$, $\beta = 1$; Red (HD): $\alpha = 1$, $\beta = 0.2$; Black (MC): $\alpha = 1$, $\beta = 1$. For these simulations, the transient time was 10^7 iterations, followed by an integration time of 10^8 iterations.

of the value of L.

It is important to note that this is not in contradiction with the results obtained in the section 'Ribosome Drop-Off Effects Depend Strongly on Codon Configuration' of the main manuscript. There, HD sequences have been shown to possess maximal resilience to drop-off when compared to other phases. Here, they are seen to have lower probability to finish translation than other phases. The former arises since the translation rate in HD remains unaffected by drop-off, since new ribosomes are initiated in sufficient numbers to compensate drop-off and thus sustaining the

queue. The latter simply reflects this fact and accounts for the reduced chance to successfully terminate translation for any given ribosome.
Moreover, the decay of $P(L)$ with L is slightly stronger in the MC phase compared to the LD phase. This is again coherent with the observation that dwell time of ribosomes are slightly higher in the MC phase, compared to the LD phase, due to more frequent steric interactions which hinder their motion.
A further interesting question is whether the translation of the longest mRNA of the *S. cerevisiae* genome remains sustainable [1]. This longest mRNA sequence corresponds to the gene YLR106C (MDN1), which has 4911 codons. Using the physiological value of the drop-off rate ($\gamma = 1.4 \times 10^{-3} s^{-1}$) and its corresponding physiological value for the initiation rate ($\alpha_\phi = 0.015 s^{-1}$) one can estimate the probability $P(L)$ to finish translation under physiological conditions: it is found to be slightly above 10%, where we have used the more realistic scenario explained in the main text, incorporating the inhomogeneous hopping rates corresponding to this particular mRNA, as well as the extended footprint of the ribosome.

3. Ribosome Drop-Off Effects Depending on Initiation Rate

Here we analyse the effect of initiation rate α on the ribosome drop-off resilience $\chi_{\tilde{\Gamma}}$. We therefore compute the resilience $\chi_{\tilde{\Gamma}}$ versus the initiation rate $\tilde{\alpha}$ for a fixed value of $\tilde{\Gamma} = 0.05$ for two different cases: (i) $\tilde{\beta} = 0.35$ and (ii) $\tilde{\beta} = 0.6$. The rest of the parameters are kept constant: $L = 1$, $N = 500$. Like that, we expect the sequence to undergo an LD-SP-HD transition in case (i), and LD-MC transition in case (ii). Figures 3, 4 show the results. The red dots indicate stochastic simulations, whereas the black lines represent the analytical results. The agreement between stochastic simulations and analytics is good in general, with the MC phase showing a slight deviation, consistent with the results presented in the section 'mRNAs with High Elongation Rates Are more Resilient to Ribosome Drop-Off' of the main manuscript.

Within the LD phase, the resilience decreases, although very slightly, as the initiation rate α is increased. However, it is constant in both the HD and MC phases, since the ribosomal current does not depend on $\tilde{\alpha}$ in those phases. Moreover, in HD phase we recover the maximal possible resilience equal to 1, and therefore, the resilience increases with $\tilde{\alpha}$ as we go through the SP (Fig. 3).

4. Estimation of Elongation Rates

We estimate the elongation rates for *S. cerevisiae* for the ribosome drop-off model following the approach taken in [1]. We assume a spatially homogenous pool of tRNAs in the cell. Let us first assign synonymous codons the same elongation rate, being proportional to the concentration of its cognate tRNA. Since not all

[1] We thank the anonymous reviewer for raising this question.

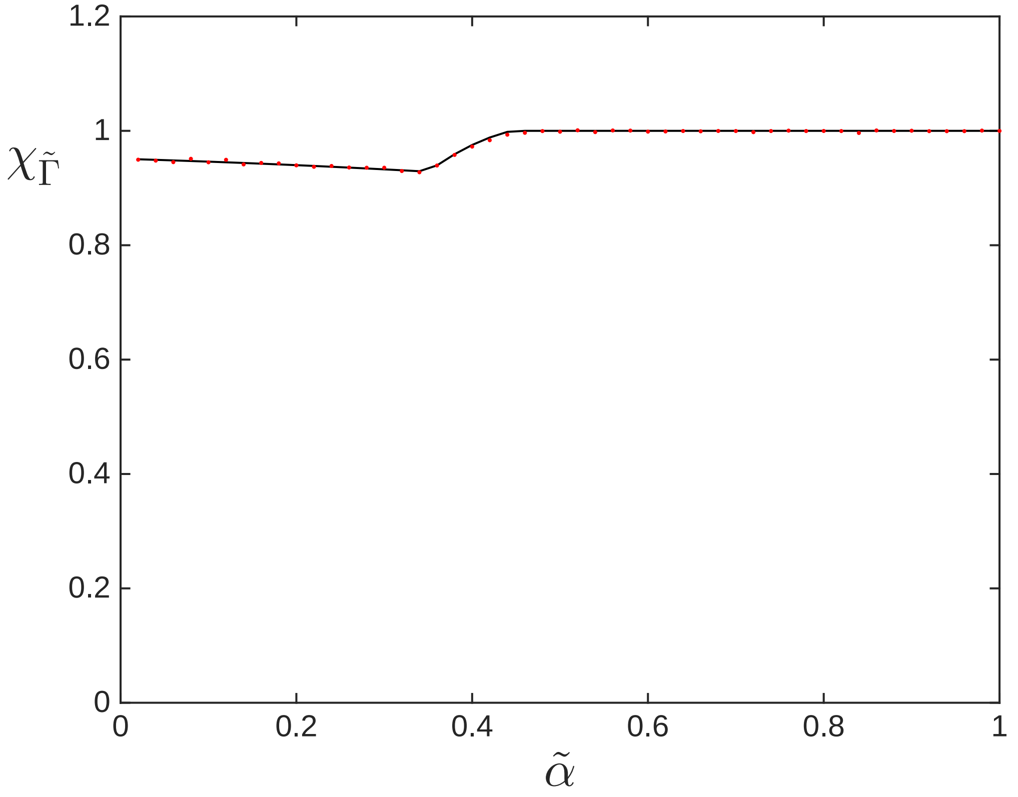

Figure 3. Ribosome drop-off resilience $\tilde{\chi}_{\tilde{\Gamma}}$ versus initiation rate $\tilde{\alpha}$ for $\tilde{\beta} = 0.35$. The lattice undergoes an LD-SP-HD phase transition. The dots indicate numerical simulations, and the solid line represents analytical results. For these simulations, the transient time was 10^7 iterations, followed by an integration time of 10^7 iterations.

tRNA abundances are available for *S. cerevisiae*, the Gene Copy Number (GCN) is taken as a proxy that indicates the proportion of different tRNAs in the cell [2]. Therefore, we start from

$$k_i = r \frac{GCN_i}{\sum_{i=1}^{41} GCN_i}, \tag{3}$$

where r is a constant of proportionality. Note that the sum in Eq (3) goes only up to 41, corresponding to the different number of tRNAs in *S. cerevisiae*.

In the following step we consider the reduction in elongation rates due to wobble base-pairing [3], so that the translation rates of codons using the G-U wobble are

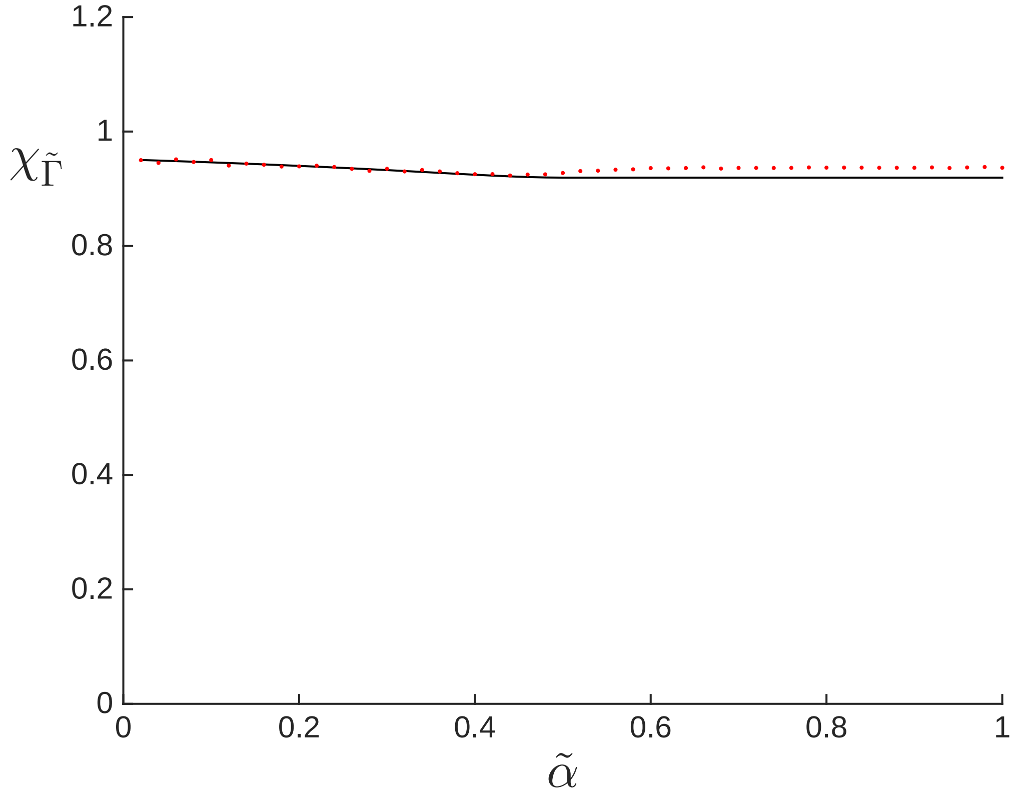

FIGURE 4. Ribosome drop-off resilience $\chi_{\tilde{\Gamma}}$ versus initiation rate $\tilde{\alpha}$ for $\tilde{\beta} = 0.6$. The lattice undergoes an LD-MC phase transition. The dots indicate numerical simulations, and the solid line represents analytical results. For these simulations, the transient time was 10^7 iterations, followed by an integration time of 10^7 iterations.

reduced by 39% compared to their G-C counterparts. Analogously, codons using the wobble I-C and codons using the wobble I-A are reduced by 36% relative to their I-U counterparts [3]. We then reduce the initially set rates accordingly. Finally, to calculate the factor of proportionality r, we impose $\langle k_i \rangle = 10$ codons/s, where $\langle \cdot \rangle$ denotes the average over all 61 codons, calculated as follows

$$\langle k_i \rangle = \sum_{i=1}^{61} k_i \frac{n_i}{n}, \tag{4}$$

where n_i denotes the total number of codons in the cell of exactly type i, and $n = \sum_{i=1}^{61} n_i$. By equating Eq (4) to 10 codons/s we can then obtain the value of r numerically. The table of the resulting elongation rates for each codon is provided in the Supplementary Tables. In this procedure the termination rate is assumed

to be fast and comparable to the highest elongation rate, and we therefore take it to be $\beta = 18.03 s^{-1}$.

5. Genome-Wide Estimation of Physiological Values of Initiation Rates

We estimate the physiological value of the initiation rate for each mRNA sequence by using yeast genome-wide experimental data of the ribosome density ρ_ϕ from [4] grown under physiological conditions. For each mRNA we identify the physiological value α_ϕ of the initiation rate, using our ribosome drop-off model by requiring $\rho(\alpha_\phi) = \rho_\phi$, i.e., we take the initiation rate to be the one which replicates the experimentally measured ribosome density, as illustrated in Fig 5 for the mRNA of YAL033W (POP5) [1].

Figure 6 shows the histogram of the genome-wide estimated values of α_ϕ. Furthermore, we have divided this histogram into four regions, from small to high α_ϕ, and performed a Gene Ontology (GO) enrichment analysis, using the software Gene Ontology Term Finder (http://go.princeton.edu/cgi-bin/GOTermFinder). P-values are computed using the hypergeometric distribution, and the Bonferroni correction is applied to take into account multiple hypothesis tests [5]. Significantly enriched GO annotations can be identified in each region of the histogram. Like that, mRNAs with $\alpha_\phi < 0.1 s^{-1}$ contain highly disproportionate number of proteins involved in biological regulation, the regulation of metabolic processes, regulation of transcription and response to stimulus, as well as proteins involved in the cell cycle, mainly located in the nucleus, protein complexes, chromosome and the membrane. In the region corresponding to initiation rates $0.1 s^{-1} < \alpha_\phi < 0.2 s^{-1}$ we find significant over-representation of proteins involved in different aspects of metabolism, such as carboxylic acid metabolic process, oxoacid metabolic process and organic acid metabolic process. In the next region, for $0.2 s^{-1} < \alpha_\phi < 0.3 s^{-1}$ GO annotations corresponding to cytoplasmic translation, metabolism, glycolysis, ATP generation from ADP are significantly over-represented, even though a large proportion of those genes are unannotated. Finally, in the last region of the histogram, for $\alpha_\phi > 0.3 s^{-1}$ most genes are unannotated. Table 1 summarises the results from the GO enrichment analysis.

These results are consistent with the results presented in the main text on the GO analysis enrichment of resilience to drop-off and its correlation with abundance of encoded proteins. From the GO analysis for the initiation rate values we see that mRNAs encoding proteins which are present at high levels in the cell (such as the ones involved in translation and glycolysis) have predominantly high initiation rates. In contrast, mRNAs encoding stress-related proteins, typically present in the cell at low levels under physiological conditions, have mainly low initiation rates.

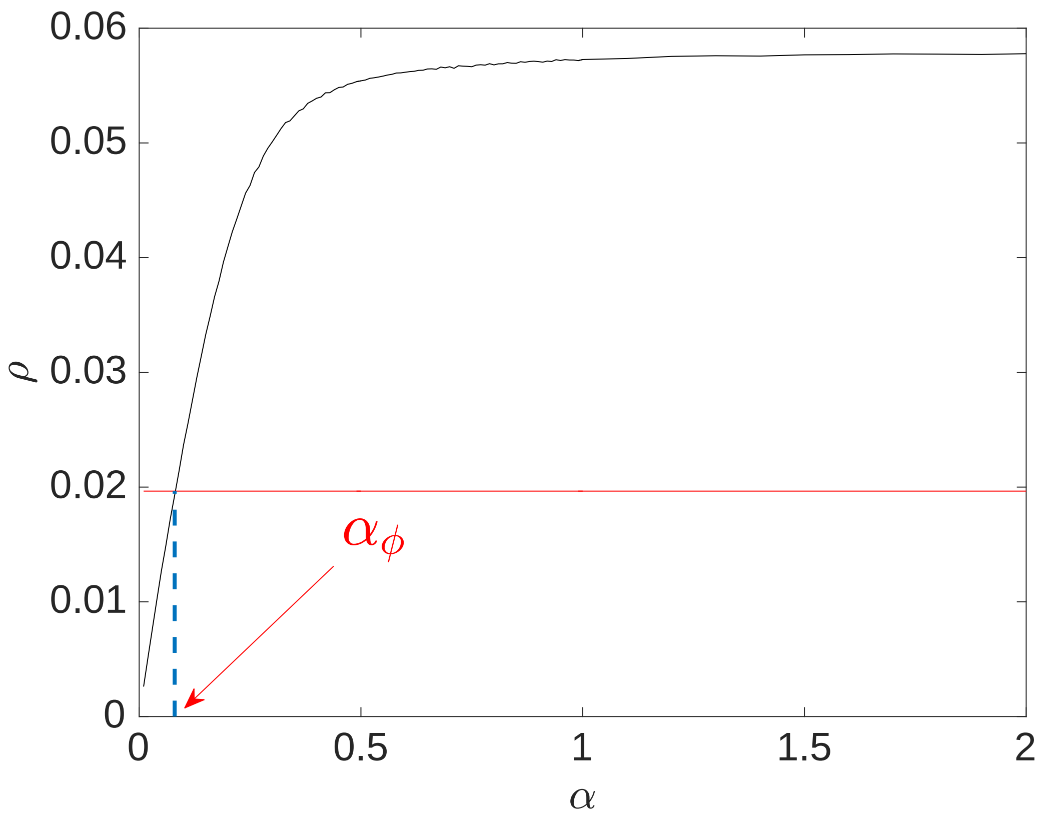

FIGURE 5. Estimation of physiological value of initiation rate α_ϕ for YAL033W (POP5). The black curve shows the simulated average ribosome density on the mRNA versus the initiation rate α. The red horizontal line shows the value of ribosome density (number of ribosomes divided by number of codons in the mRNA) measured under physiological conditions [4]. The value of α_ϕ is given by the value of α at which the simulated ribosome density coincides with the measured one.

The table of α_ϕ values for each mRNA, as well as the complete results from this GO analysis are provided in the Supplementary Tables.

REFERENCES

[1] Ciandrini L, Stansfield I, Romano MC. Ribosome Traffic on mRNAs Maps to Gene Ontology: Genome-wide Quantification of Translation Initiation Rates and Polysome Size Regulation. PLoS Computational Biology 2013;9:e1002866. http://doi.org/10.1371/journal.pcbi.1002866

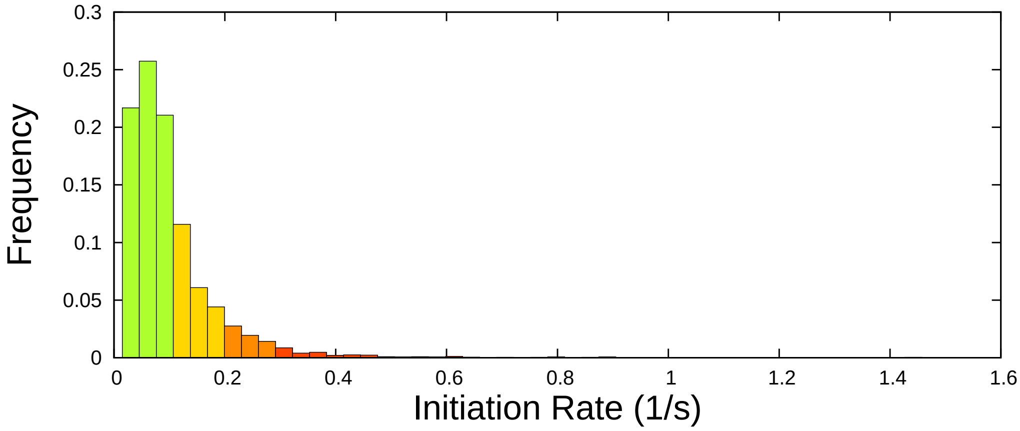

FIGURE 6. Histogram of physiological values of the initiation rate α_ϕ for the *S. cerevisiae* genome. The colours of the different regions correspond to the following α_ϕ intervals: green: $\alpha_\phi < 0.1s^{-1}$; yellow: $0.1s^{-1} < \alpha_\phi < 0.2s^{-1}$; orange $0.2s^{-1} < \alpha_\phi < 0.2s^{-1}$, and red: $\alpha_\phi > 0.3s^{-1}$.

[2] Percudani R, Pavesi A, Ottonello S. Transfer RNA gene redundancy and translational selection in Saccharomyces cerevisiae. Journal of Molecular Biology 1997; 268:322-330.
[3] Gilchrist MA, Wagner A. A model of protein translation including codon bias, nonsense errors, and ribosome recycling, Journal of Theoretical Biology 2006; 239, Issue 4: 417-434.
[4] MacKay VL, Li X, Flory MR, Turcott E, Law GL, et al. (2004) Gene expression analyzed by high-resolution state array analysis and quantitative proteomics. Molecular & Cellular Proteomics 3: 478-489.
[5] Boyle EI, Weng S, Gollub J, Jin H, Botstein D, Cherry JM, Sherlock G. GO::TermFinder open source software for accessing Gene Ontology information and finding significantly enriched Gene Ontology terms associated with a list of genes. Bioinformatics 2004; 20 (18): 3710-3715. doi: 10.1093/bioinformatics/bth456

Region	α_ϕ range	GO term	P-value
green	$[0, 0.1)$	biological regulation	5.11e-93
		regulation of metabolic process	5.44e-49
		regulation of transcription, DNA-templated	1.74e-41
		response to stimulus	1.61e-40
		cell cycle	1.11e-38
yellow	$[0.1, 0.2)$	single-organism metabolic process	1.75e-17
		small molecule metabolic process	8.69e-16
		carboxylic acid metabolic process	2.77e-13
		oxoacid metabolic process	5.75e-13
		organic acid metabolic process	7.35e-13
orange	$[0.2, 0.3)$	unannotated	3.92e-14
		cytoplasmic translation	2.95e-11
		ADP metabolic process	1.81e-05
		glycolytic process	0.00112
		ATP generation from ADP	0.00112
red	$(0.3, 1]$	unannotated	1.91e-15

Table 1. Table summarising the results for Gene Ontology (GO) enrichment analysis for the 4 different sections of the histogram of the initiation rate values (Fig 6) obtained for the *S. cerevisiae* genome.
